# Molecular Architectures of Trimeric SIV and HIV-1 Envelope Glycoproteins on Intact Viruses: Strain-Dependent Variation in Quaternary Structure

**DOI:** 10.1371/journal.ppat.1001249

**Published:** 2010-12-23

**Authors:** Tommi A. White, Alberto Bartesaghi, Mario J. Borgnia, Joel R. Meyerson, M. Jason V. de la Cruz, Julian W. Bess, Rachna Nandwani, James A. Hoxie, Jeffrey D. Lifson, Jacqueline L. S. Milne, Sriram Subramaniam

**Affiliations:** 1 Laboratory of Cell Biology, Center for Cancer Research, National Cancer Institute, NIH, Bethesda, Maryland, United States of America; 2 AIDS and Cancer Virus Program, SAIC-Frederick, Inc., National Cancer Institute-Frederick, Frederick, Maryland, United States of America; 3 Department of Medicine, University of Pennsylvania, Philadelphia, Pennsylvania, United States of America; Institut Pasteur, France

## Abstract

The initial step in target cell infection by human, and the closely related simian immunodeficiency viruses (HIV and SIV, respectively) occurs with the binding of trimeric envelope glycoproteins (Env), composed of heterodimers of the viral transmembrane glycoprotein (gp41) and surface glycoprotein (gp120) to target T-cells. Knowledge of the molecular structure of trimeric Env on intact viruses is important both for understanding the molecular mechanisms underlying virus-cell interactions and for the design of effective immunogen-based vaccines to combat HIV/AIDS. Previous analyses of intact HIV-1 BaL virions have already resulted in structures of trimeric Env in unliganded and CD4-liganded states at ∼20 Å resolution. Here, we show that the molecular architectures of trimeric Env from SIVmneE11S, SIVmac239 and HIV-1 R3A strains are closely comparable to that previously determined for HIV-1 BaL, with the V1 and V2 variable loops located at the apex of the spike, close to the contact zone between virus and cell. The location of the V1/V2 loops in trimeric Env was definitively confirmed by structural analysis of HIV-1 R3A virions engineered to express Env with deletion of these loops. Strikingly, in SIV CP-MAC, a CD4-independent strain, trimeric Env is in a constitutively “open” conformation with gp120 trimers splayed out in a conformation similar to that seen for HIV-1 BaL Env when it is complexed with sCD4 and the CD4i antibody 17b. Our findings suggest a structural explanation for the molecular mechanism of CD4-independent viral entry and further establish that cryo-electron tomography can be used to discover distinct, functionally relevant quaternary structures of Env displayed on intact viruses.

## Author Summary

HIV and SIV contact and infect target T-cells following the binding of trimeric Env spikes displayed on the viral membrane with cellular receptors. The conformational changes in trimeric Env that are triggered by the interaction between trimeric Env and cell surface receptors lead ultimately to fusion of the viral and cell membranes and delivery of the viral core into infected cells. Knowledge of the molecular structures of trimeric Env at different stages of virus-cell contact is therefore of fundamental interest for defining viral entry mechanisms and vaccine design. Cryo-electron tomography is a powerful structural tool to determine the structures of viral spikes when they are present on the surface of intact virions. Using this approach, we have determined the molecular structures of several SIV and HIV-1 strains, including an SIV strain that does not require cell surface receptor CD4 for entry and infection. Our results represent the first experimental demonstration that strain differences can result in distinct unliganded spike conformation as displayed on the surface of intact virions. The differences in structure between the different strains correlate with functional differences displayed by the viruses, and suggest a novel molecular explanation for the mechanism of CD4-independent viral entry.

## Introduction

About 2.5 million individuals are newly infected with human immunodeficiency virus (HIV) each year, and over 2 million deaths result annually from HIV/AIDS (http://www.unaids.org). HIV-1 and the closely related simian immunodeficiency virus (SIV) bind to target cells by the interaction of trimeric envelope glycoprotein spikes (Env), a heterodimer of a transmembrane glycoprotein (gp41) and a surface glycoprotein (gp120) [1], with CD4 and a co-receptor (CCR5 or CXCR4) [2]. Understanding of the molecular structure of trimeric Env on infectious virus particles before and after contact with the T-cell surface is fundamental to the informed design of immunogens to elicit broadly neutralizing antibodies and for deciphering the detailed molecular mechanisms underlying HIV infection [3], [4]. Presently, no atomic resolution structures are available for trimeric Env in any conformational state, although there are several sets of coordinates available from X-ray crystallography for the truncated core of monomeric gp120 in unliganded [5] and liganded forms [6], [7], [8].

Recent advances in cryo-electron tomography to obtain 3D density maps from pleiomorphic biological structures provide new methods to tackle the challenge of describing the structure of trimeric Env as displayed on infectious viruses under near-native conditions [9]. Starting from a series of tilted projection images of plunge-frozen viruses, tomograms that capture the distribution of density on the surface and interior of the virus can be determined. In general, the resolution that can be obtained in a tomogram of a single virus is barely enough to discern molecular shapes because images are recorded at the lowest possible electron doses in order to minimize damage from electron irradiation of the sample. However, by extracting subvolumes corresponding to each trimeric spike, and accounting properly for the missing wedge [10] of data that is inherent to electron tomography, 3D classification and averaging can be used to obtain density maps at signal-to-noise ratios that are sufficiently high for molecular interpretation [9], [10], [11], [12].

Cryo-electron tomographic studies have been recently used to obtain density maps for trimeric Env on both HIV-1 and SIV [13], [14], [15], [16] (see also Figure S1). Analysis of HIV-1 virions resulted in structures of trimeric Env in unliganded, b12-antibody neutralized and sCD4/17b-antibody liganded states at ∼20 Å resolution and in a working model for structural changes in trimeric Env that occur upon engagement of the CD4 receptor on a target cell [15]. The validity of the experimental and computational procedures used to obtain the density maps of trimeric HIV-1 gp120 were first established [10] by determining structures of protein complexes with known structures at atomic resolution and of gp120-antibody complexes. Previous tomographic studies of trimeric SIV Env have only focused on unliganded Env and have been controversial. Density maps and derived molecular models reported for trimeric Env displayed on SIVmac239 by Zhu et al. [13] and SIVmneE11S by Zanetti et al. [14] differ significantly from each other and from that reported in our earlier studies of unliganded HIV-1 BaL [10]. It has remained unclear whether the disagreements in the density maps reflect genuine differences in structures of trimeric Env between the different viral isolates studied or originate largely from variations in the strategy and methods used for structure determination.

The choice of gp120 atomic coordinates chosen to interpret the results from tomography is also controversial, as the available atomic coordinates of monomeric SIV gp120 in unliganded [5], and monomeric HIV-1 gp120 in various liganded and antibody-bound states [6], [7], [8] display significant differences, especially in the inner domain. To address this controversy, we have carried out electron tomographic studies of SIVmneE11S and SIVmac239 from the same purified virus preparations used in the previous conflicting reports [13], [14], using the computational procedures established in our previous work [10], [15] on trimeric HIV-1 BaL Env. Here, we present both biochemical and computational evidence to validate the fits of gp120 to the density maps derived by cryo-electron tomography and extend the analysis to a CD4-independent SIV strain where we show that trimeric Env is displayed in a different quaternary conformation.

## Results

### Structures of unliganded trimeric SIV Env

**Figure 1:**
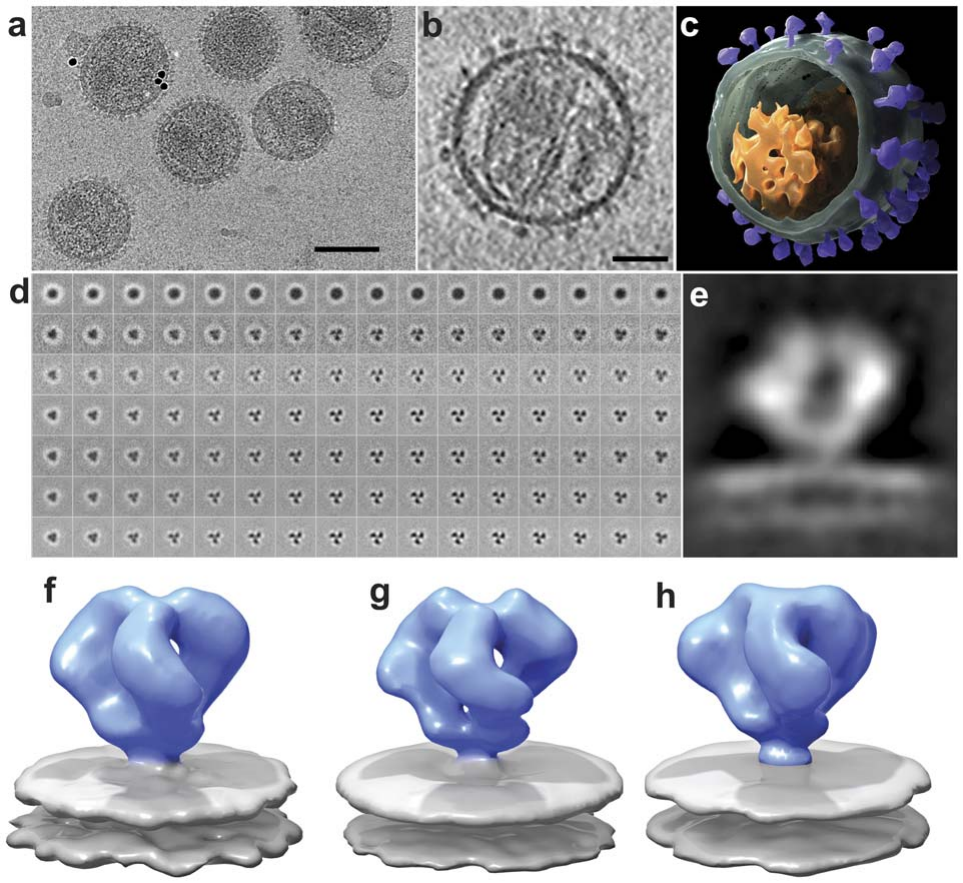
Cryo-electron tomography and molecular architecture of trimeric SIV Env. (a) Low-dose (12 electrons/Å^2^) projection image recorded from purified SIVmneE11S virions plunge-frozen directly in physiological buffer. The dark spots are 10 nm-sized gold particles that serve as fiducial markers for tilt series alignment. (b) Slice through reconstructed cryo-electron tomograms of a single SIVmneE11S virus. The image was obtained by averaging 6 slices from a 4×4 binned tomogram and is ∼10 nm thick. The viral core and surface spikes can be clearly visualized in the image. (c) 3D rendering of the virion shown in (b), segmented to highlight the viral membrane (green), core (orange) and abundant surface spikes (purple). (d) Results from classification and 3D averaging of ∼4000 3D volumes of SIVmneE11S showing improvement from the first round (second row), to the final round (bottom row) of image classification (see Figure S2 for expanded version of this figure panel). Early stages of classification clearly show classes with inherent 3-fold symmetry, and typically at the fourth iteration, 3-fold symmetry was imposed. At the end of each iteration, the classes that showed the most clearly delineated features in all regions of the spike were selected and combined for use as a reference for the next round. The progression of images from left to right represent successive slices (4.1 Å thickness) from bottom to top of Env through the density map of averaged classes that was used as a reference for the next iteration. The subset of slices shown spans a region of ∼65 Å between the gp120/gp41 interface to the apex of gp120. (e) 2D density profile from a slice through the density map for SIVmneE11S derived from classification and 3D averaging of ∼4000 spikes. (f-h) Perspective views of density maps for SIVmneE11S (f), SIVmac239 (g) and HIV-1 BaL (h) Env shown as isosurface representations with envelope glycoprotein ectodomain shown in blue and viral membrane shown in gray. Scale bars are 100 nm in panel (a) and 35 nm in panel (b).

A low-dose projection image recorded at zero tilt from a specimen of plunge-frozen SIVmneE11S is shown in Figure 1a. The viral membranes are visible, but the surface spikes can be barely discerned. Much more detail is evident in a tomogram constructed from a series of images spanning a tilt range of ±65° where the viral envelope and individual spikes can be easily detected (Video S1, S2). Inspection of slices through the tomogram (Figure 1b) provides an indication of the improvement in image quality compared to the single projection image in Figure 1a. The tomographic volumes can be visualized either as an image stack (Video S1, S2), or as a segmented rendering (Figure 1c). To obtain a density map for each trimeric Env, typically 3000–4000 spikes present on the surface of reconstructed virions were selected using automated procedures and subjected to 3D alignment, classification and 3D averaging (Figure 1d) as described previously [10] to obtain profiles of 3D density distribution (see Figure S2 for expanded version of Figure 1d). The maps can also be visualized as a cross-sectional slice through the density to view the overall structural profile of Env (Figure 1e). The highest densities and highest signal-to-noise ratios are observed in the gp120 region and at the putative gp120/gp41 interface, indicating that these are the best-defined regions of the structure. There is lower density and a lower signal-to-noise ratio in the region immediately above the membrane, corresponding to the gp41 stalk, implying that there is less scattering power (i.e. protein mass) and/or possibly greater disorder in this region relative to the gp120 region. The methods that we have used for image classification and alignment and for missing wedge correction minimize the possibility that these less defined regions of the map lead to artifacts in the final averaged map. The maps for SIVmneE11S and SIVmac239 strains, shown as isosurface representations (Figures 1f and 1g, respectively) establish that the two spikes are similar in shape, each being composed of a propeller-shaped region with three gp120 blades connected both at the apex and at the base, corresponding to the gp120/gp41 interface. This architecture closely resembles that reported previously for unliganded HIV-1 Env at ∼20 Å resolution (Figure 1h; [15]).

### Comparison with earlier analyses of trimeric Env

Our finding that the gp41 stalk connecting the gp120/gp41 interface to the membrane is compact is in agreement with this feature of the SIVmneE11S Env map reported by Zanetti et al [14] and our earlier study of HIV-1 BaL Env, but at variance with the reports by Zhu et al. for SIVmac239 [13] and HIV-1 BaL Env [16] where the stalk region was separated into the legs of a tripod that are 70 Å apart (Figure S1). In addition to discrepancies in the overall shape of the maps, there are significant differences in the molecular interpretation of the spike architectures, derived by fitting X-ray crystallographic atomic coordinates for monomeric gp120 into the density maps obtained using cryo-electron tomography. There are multiple X-ray coordinates for truncated gp120 monomers, in unliganded, as well as sCD4-liganded or antibody-bound forms [6], [7], [8]. Of these, the coordinates for monomeric, unliganded SIV gp120 (PDB ID: 2BF1; [5]) might be considered a natural choice to obtain a molecular interpretation of the unliganded HIV-1 and SIV Env trimers since these are the only published coordinates for unliganded SIV or HIV-1 gp120. The two previously reported density maps for trimeric SIV Env were interpreted in terms of these coordinates using manual fitting. Zhu et al. [13] oriented the monomeric gp120 coordinates in the map so that the V1 and V2 loops are located at the base of the spike. Zanetti et al. [14] presented two alternate interpretations in which the V1 and V2 loops also localized to the base of the spike, but with substantial differences in the predicted positions of the V3 loop. Each of these models also differed from the theoretical model proposed by Chen et al. [5] which also had the V1/V2 loop at the base of the spike (Figure S3c, S3d), but with the V3 loops of the three gp120 monomers located at the apex of the spike. Thus, in addition to the differences in overall shape of the density maps, these earlier models derived by electron tomography also provided radically different interpretations for the molecular structure of trimeric SIV Env. The differences in the final outcome can only arise from four possible sources: (i) procedures and instrumentation used for data collection, (ii) the computational procedures used to average individual tomographic spike subvolumes to arrive at the final density map, (iii) the choice of gp120 coordinates used to interpret the maps and (iv) the procedures used to fit the coordinates into the density map. Of these, the first source can be excluded because the differences in methods for data collection between the two previously published tomographic analyses [13], [14] and the present work are not expected to alter the profile of the density maps, with only minor differences such as pixel size (5.5 Å vs. 4.1 Å) and underfocus values (4 to 6 µm vs. 1.5 to 2.5 µm). The remaining three possibilities and a description of the procedure for choice and fitting of coordinates into the density maps are discussed below (see also Figures S2, S3, S4, S5, S6).

### Choice of coordinates and determination of fit

The determination of density maps for the complex of trimeric Env with b12 and sCD4/17b was a critical element of our earlier analysis with antibody-bound HIV-1 spikes because the X-ray structures available for the binary [8] and ternary [7] complex, respectively, provided unambiguous tests of map quality and interpretation [15]. Since there are no obvious constraints such as the presence of complexed b12 or sCD4 for obtaining a molecular interpretation of unliganded SIV/HIV-1 Env density maps, the fitting is based on the fit with the gp120 coordinates alone. All of the available gp120 coordinates include only ∼60% of gp120 polypeptide mass, and none of the gp41 ectodomain (∼20 kD), which makes the fit of these coordinates into the density map challenging. Placing any of these gp120 coordinates into the map by manual fitting as in the work of Zhu et al. [13] or Zanetti et al. [14] is non-quantitative, since the outcome is based on subjective evaluation of the map. The automated fitting procedures we use employ correlation coefficient-based maximization, providing an objective way of obtaining the fits. Further details about the fitting procedures are also available publicly through the website of the publicly distributed software package UCSF Chimera (http://www.cgl.ucsf.edu/chimera/hiv2009/).

The three possible sets of atomic coordinates that are available as starting points to carry out the automated fitting are the structure reported for unliganded SIV gp120 core (2BF1), the structure of HIV-1 gp120 from the complex with b12 (2NY7), or the structure of HIV-1 gp120 from the complex with sCD4/17b (1GC1). The automated fitting procedures, identical to those used in our previous work with HIV-1, result in a single solution when the HIV-1 gp120 coordinates are used, and no clear solution and lower correlation coefficients (indicative of a poor fit) when the 2BF1 SIV gp120 coordinates are used in any orientation. Comparison of the fit obtained from our work using 1GC1 coordinates (Figures S3a, S3b) with the theoretical model (Figures S3c, S3d) proposed by Chen et al. [5] based on their crystallographic structure of unliganded, truncated SIV gp120 shows that the two differ dramatically in terms of the inferred locations of the V1/V2 loop regions. In the preferred fits with 1GC1 coordinates, the V1/V2 loop regions are at the apex of the spike, while the model proposed by Chen et al. [5] places the V1/V2 loop regions closer to the base of the spike (Figures S3c, S3d).

These differences can be visualized more clearly in Figure S4. The first column in Figure S4 shows density maps calculated from each of these gp120 coordinates to a resolution of 20 Å, which illustrates the appearance of each coordinates' density at this resolution when rendered as an isosurface. The gp120 density profiles obtained from the coordinates of the sCD4/17b complex (Figure S4a) and the b12 complex (Figure S4b) are rather similar when viewed at ∼20 Å resolution, even though there are important differences in their atomic resolution structures. However, both of these HIV-1 gp120 structures differ significantly from the conformation reported for monomeric, unliganded, SIV gp120 (Figure S4c). When each of these coordinates are fitted into the SIVmneE11S map we present here, it is the HIV-1 gp120 coordinates determined for the complexes with sCD4/17b (Figures S4d, S4g) or b12 (Figures S4e, S4h) that show a visually better fit rather than the coordinates reported for the unliganded SIV gp120 core (Figures S4f, S4i). The estimated position of the V1/V2 loops, based on the location of the truncated loop in the coordinates indicated by red spheres, also varies between the 3 sets of gp120 coordinates. The fits of both sets of HIV-1 gp120 coordinates situate the V1/V2 loop region with excellent correspondence to a region of unassigned density at the apex of the spike (Figure S4d, S4e, S4g, S4h). In contrast, the estimated location of this loop in the unliganded SIV gp120 coordinates (Figure S4f, S4i) is not consistent with the observed architecture of the spike, falling in a region where there is no unassigned density. All three sets of coordinates also have significant deletions in the N and C-terminal regions that reside at the base of the spike.

In order to further compare the different fits, we extended our analysis to use a stringent “geometric fitting” criterion [15] that evaluates the fit over a range of density thresholds (Figure S5). In cryo-electron microscopy, choice of the “right threshold” to represent density maps generally depends on the size of the protein, and on an effective “temperature-factor”, which describes the resolution-dependent fall-off in image amplitudes. The reason why the use of correlation coefficients alone can lead to false confidence in fit quality is because the density map is influenced by both parameters, and there is no *a priori* way to know these parameters precisely. Analyzing the map over a complete range of thresholds is thus a thorough and transparent way to illustrate map quality, and provides a better way to assess quantitative fitting coefficients. For this purpose, the fit of the monomeric HIV-1 and SIV gp120 coordinates as shown in Figures S4b, S4e, or S4f was kept fixed, and the threshold values for map visualization was progressively varied (Figure S5). At each threshold value, the proportion of atoms that fell outside the map contour was calculated. We note that the coordinate fits are to the density map and not the threshold, and therefore are identical across all thresholds. Compared to the fits obtained using 1GC1 or 2NY7 coordinates, a higher proportion of atoms are distributed outside the map contour at every threshold when 2BF1 coordinates are used to carry out the fit in the orientation roughly corresponding to that suggested by Chen et al. [5]. These results provide further validation regarding the choice of coordinates used for fitting by giving a quantitative estimate of the number of atoms that are included within the map over a range of density thresholds. These calculations do not include contributions from the V1/V2 loop which are absent in the coordinates; if this loop is included, the improved fit of the HIV-1 coordinates to the map would be further accentuated. Inclusion of the stump of the loop present in the 1GC1 coordinates also makes no difference to the fits. In summary, the molecular model for trimeric SIV and HIV-1 envelope glycoproteins derived from published coordinates for liganded monomeric HIV-1 gp120, but not SIV gp120 is consistent with the density maps we have obtained from native virions. The most likely explanation is that the three-dimensional crystals used to determine the structure of the truncated SIV gp120 core may have captured a conformation of gp120 that is different from the conformation in the native trimer.

### Independent validation of the location of loops using loop-deleted HIV-1 Env

**Figure 2:**
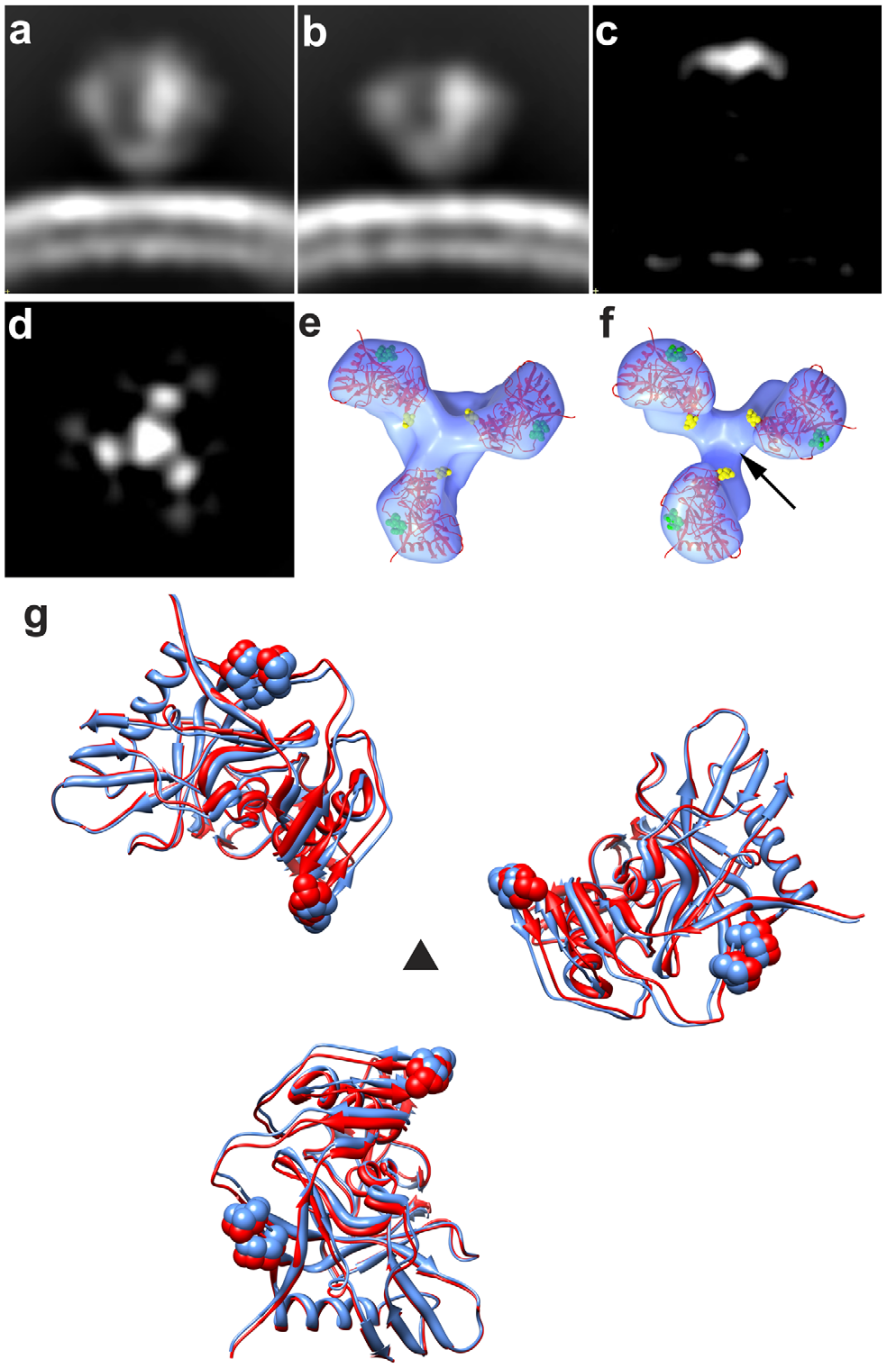
Structural analysis of full-length and V1/V2 loop-deleted HIV-1 R3A trimeric Env. (a, b) Side view of raw density projections for full-length trimeric HIV-1 R3A Env (a) and the V1/V2-loop deleted HIV-1 R3A mutant (b) showing the loss of density at the apex in the mutant. This is confirmed by location of the peak in the difference density projections between full-length and loop deleted Env (c, d), shown both as a side view (c) and as a top view (d). (e, f) Top views of fitted density maps rendered as isosurfaces for wild-type and V1/V2 loop-deleted mutants, respectively, of the HIV-1 R3A strain. The gp120 coordinates (1GC1) are shown as red ribbons and were fitted using automated procedures into the density maps. The black arrow in (f) points to the location of the missing density in the V1/V2 loop-deleted variant, and residues in stump of the V1/V2 and V3 loops in the coordinates are highlighted in yellow and in green spheres, respectively. (g) Superposition of fitted coordinates for the gp120 trimer in the full-length and V1/V2 loop-deleted viruses with fits to the wild-type map in red and the mutant in blue, with the black triangle representing the 3-fold symmetry axis.

The V1 and V2 loop regions together represent ∼15–20% of the overall polypeptide mass of gp120. The earlier studies with b12-complexed HIV-1 BaL [15], as well as the detailed fitting exercises described in Figure S4 provide strong evidence that the V1 and V2 loop regions are located at the apex of the spike. As a further stringent test of this assignment, we took advantage of the availability of a previously reported V1/V2 loop-deleted variant of the HIV-1 R3A strain [17]. For this purpose, a matched pair of full-length and loop-deleted viruses of the R3A strain were prepared in parallel using the same cell system, purified in parallel under the same conditions, and analyzed by cryo-electron tomography. The quaternary structure of Env from the R3A strain (Figure 2a) is essentially the same as that previously reported in our earlier studies of HIV-1 BaL [15]. Comparison of trimeric Env structures from V1/V2 loop-deleted HIV-1 R3A (Figure 2b) with the wild-type R3A shows missing density in the raw map of the deletion mutant at the predicted location and is confirmed in the difference map (Figures 2c–d). These results establish unequivocally that the V1/V2 loops must be located at the top of the trimeric spike. Further, automated fitting of the density maps with 1GC1 HIV-1 gp120 coordinates results in molecular structures for trimeric Env in wild-type and V1/V2 loop-deleted variants of HIV-1 R3A that are closely comparable to each other (Figures 2e–g), and to that reported for HIV-BaL [15].

### Molecular architecture of trimeric SIV Env

**Figure 3 ppat-1001249-g003:**
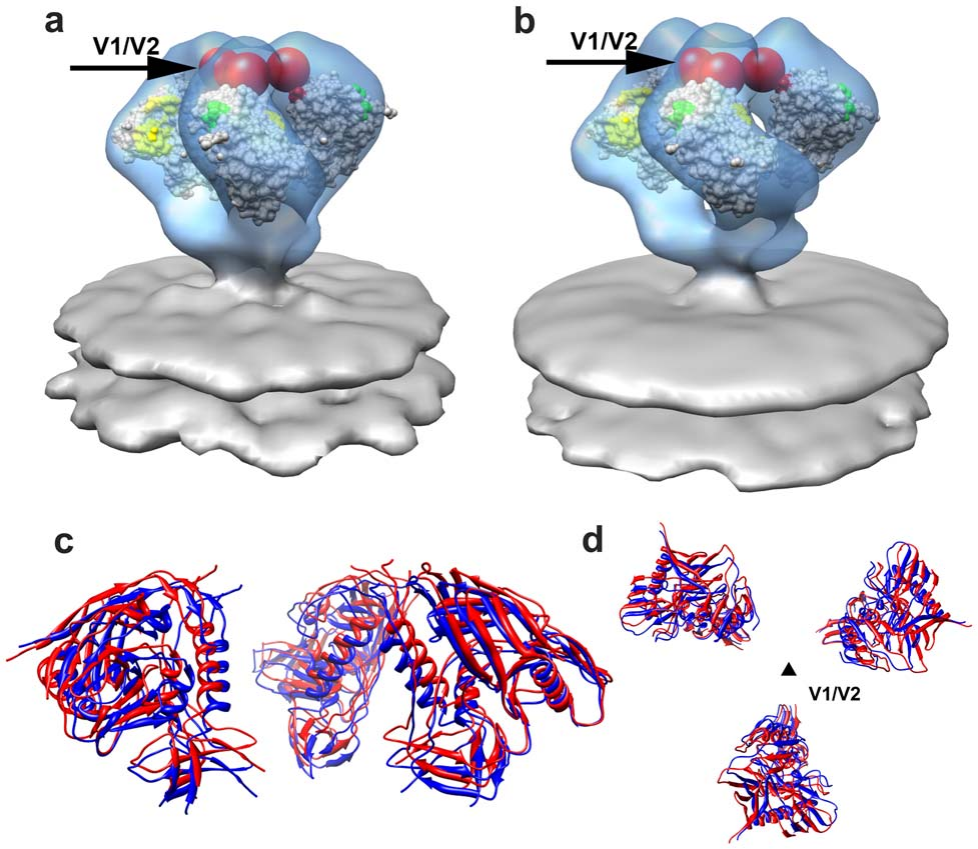
Molecular architecture of trimeric SIV Env. (a, b) Perspective views of gp120 monomer coordinates (1GC1) fit into the Env density maps obtained from SIVmneE11S (a) and SIVmac239 (b) using automated procedures. Maps are rendered as transparent isosurfaces with the grey space-filling model of gp120 colored green at base of the V3 loop with the CD4-binding site shown in yellow. The red spheres indicate location of the ∼90 V1/V2 residues of the loop (black arrows) missing in the gp120 coordinates. (c, d) Superposition of the fitted gp120 coordinates for SIVmneE11S (blue ribbons) and SIVmac239 (red ribbons) shown in front (c) view and rotated 90° around the x-axis to display the top (d) view, with the black triangle representing the 3-fold symmetry axis.

Using the principles for fitting gp120 coordinates described above, we obtained a molecular model for trimeric Env from SIVmneE11S and SIVmac239 (Figures 3a and 3b, respectively). Visualization of the fits of the gp120 coordinates to the map identifies the locations of the V1/V2 loop region absent in the X-ray coordinates, and the gp41 ectodomain. The fitted gp120 trimers for SIVmneE11S and SIVmac239 display similar architectures, with differences in orientations and total displacement of the fitted gp120 molecules that are within ∼10° and ∼10 Å, respectively. In turn, both are similar to the structure of trimeric HIV-1 BaL [15].

**Figure 4 ppat-1001249-g004:**
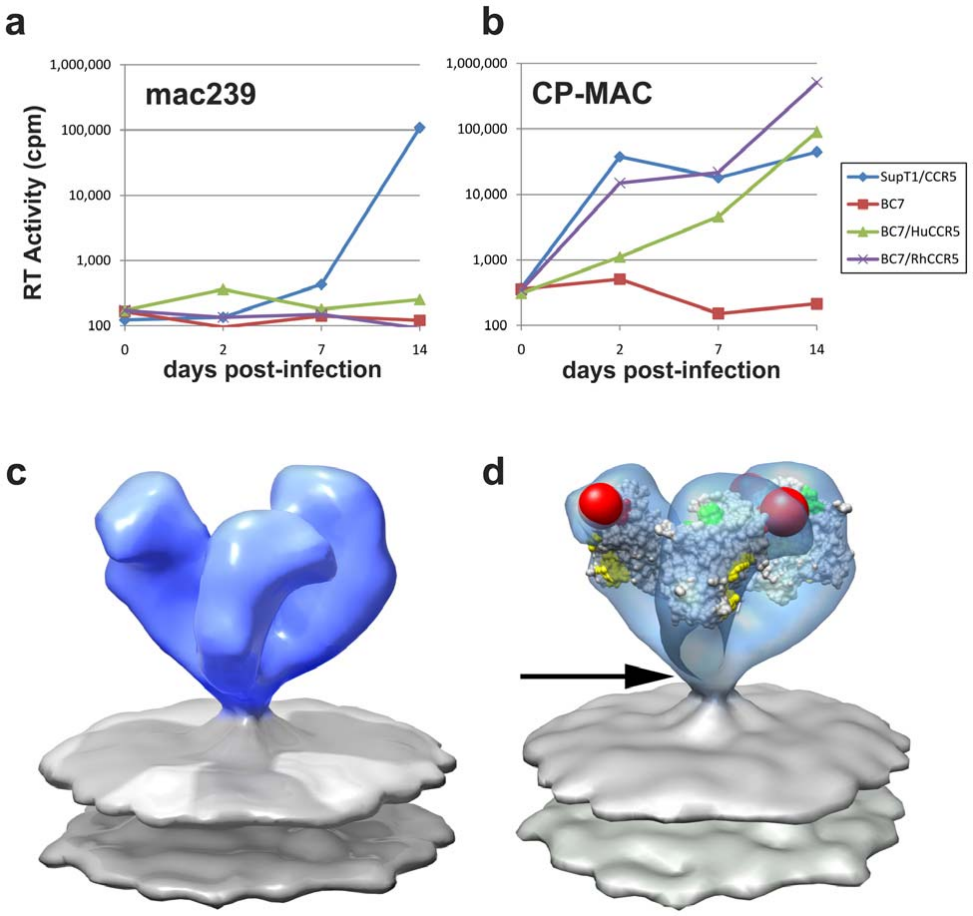
Molecular architecture of constitutively open trimeric SIV Env from the CD4-independent CP-MAC strain. (a,b) Demonstration of CD4-independent viral entry by SIV CP-MAC virus based on infectivity as determined on CD4^+^ (SupT1) or CD4^−^ (BC7) cells bearing human or rhesus CCR5 for SIVmac239 (a) or SIV CP-MAC (b). Infectivity was determined by reverse transcriptase assays of culture supernatant and confirmed by immunofluorescence assays of cells for SIV-p27^gag^ (Figure S8). (c) Perspective view of density map of Env at ∼20 Å resolution for SIV CP-MAC shown as an isosurface representation. (d) Automated fit of HIV-1 gp120 monomer coordinates (1GC1) into the density map with the CD4 binding site and the base of the V3 loop highlighted in yellow and green, respectively. Absent in the gp120 coordinates are ∼90 residues in the V1/V2 loops, and ∼100 residues in the N- and C-termini, close to the gp120/gp41 interface. The missing V1/V2 loop is represented by the red sphere and the location of the gp120/gp41 interface is indicated by the black arrow.

We extended the investigation of trimeric SIV spike structure to a third strain, SIV CP-MAC, a cytopathic SIV variant derived by serial passaging of SIVmac BK28 on CCR5-negative SupT1 cells [18], [19], [28]. SIV CP-MAC is of interest because its Env has been reported to exhibit CD4-independent utilization of rhesus CCR5 on Env-pseudotyped reporter particles [20], in contrast to viruses such as SIVmac239 where infection is CD4-dependent. Using BC7, a CD4-negative variant of the T-cell line SupT1 [19], [30] that was engineered to stably express human or rhesus CCR5, we confirmed that CP-MAC, but not SIVmac239, could efficiently infect these cells in the absence of CD4 (Figures 4a, 4b, S8). Density maps for trimeric Env from SIV CP-MAC show that Env on this virus is present in a structurally distinct “open” conformation (Figure 4c) in which the three blades of the propeller are splayed outwards at the apex of the spike in striking contrast to SIVmneE11S and SIVmac239 that, display Env in a “closed” conformation (Figure 1f, 1g respectively) with the gp120 propeller blades pointed inwards towards the apex.

**Figure 5:**
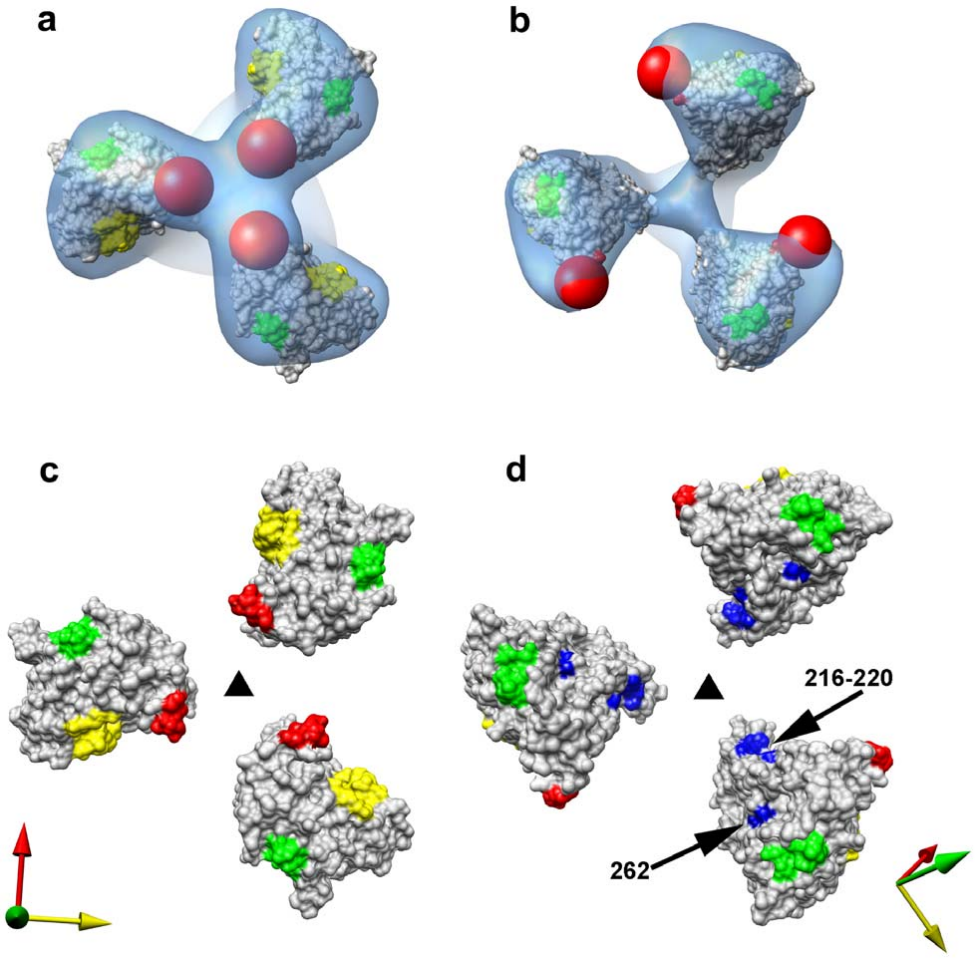
Closed and open states of trimeric SIV Env. (a, b) Top views of the density maps of (a) closed (SIVmneE11S) and (b) open (SIV CP-MAC) states fitted with gp120 coordinates. The missing V1/V2 loop is represented by the red sphere to highlight the dramatic difference in location of the loop between the two states. (c, d) Top view of the molecular surfaces of gp120 trimers in (c) closed (SIVmneE11S) and (d) open (SIV CP-MAC) states, highlighting selected residues (216–220, 262, shown in blue) that are conserved across most SIV and HIV-1 Env sequences, which are buried in the closed state, but exposed in the open state. Residues in the CD4 binding site and in the stem of the V1/V2 and V3 loops are shown in yellow, red and green, respectively. The coordinate system in each panel provides an aid to visualizing the rotation of each gp120 monomer in the transition from the closed to open state. Filled dark triangle indicates location of the 3-fold symmetry axis.

Comparison of the fits of gp120 to maps of the closed (Figures 5a, 5c) and open states (Figure 4d, 5b, 5d) of trimeric Env reveal that relative to the fits obtained for the closed conformation, each of the gp120 monomers in the open SIV CP-MAC Env displays an in-plane rotation of ∼50° coupled with an out-of-plane rotation of ∼20° and upward displacement of ∼10 Å (Figure 5). In this open conformation, the V1/V2 loop is rotated away from the apex of the spike towards the periphery, the CD4 binding site is also similarly rotated, and the V3 loop is closer to the apex of the spike, poised for binding the co-receptor. Notably, the quaternary arrangement of gp120 in trimeric SIV CP-MAC Env is in essentially the same “open” conformation previously observed for trimeric gp120 on CD4/17b complexed HIV-1 BaL (PDB ID: 3DNO; [15]), which represents a conformational state achieved prior to viral entry but after binding to cellular CD4. Several residues, including those that are highly conserved across most SIV and HIV sequences, which are buried in the closed state (Figure 5c) become exposed in the constitutively open state (Figure 5d). It is of particular interest that a swath of residues which becomes exposed upon opening at the apex of the spike include those in the vicinity of the V3 loop region, spanning regions that may potentially represent co-receptor-interactive sites. The discovery of the constitutively open conformation in SIV CP-MAC suggests that the formation of this open conformation is the underlying molecular mechanism by which this CD4-independent virus enters cells lacking CD4, and further investigation is necessary to determine whether other immunodeficiency viruses with similar entry requirements share this molecular architecture.

### Structure of 7D3-antibody bound SIV CP-MAC Env

**Figure 6:**
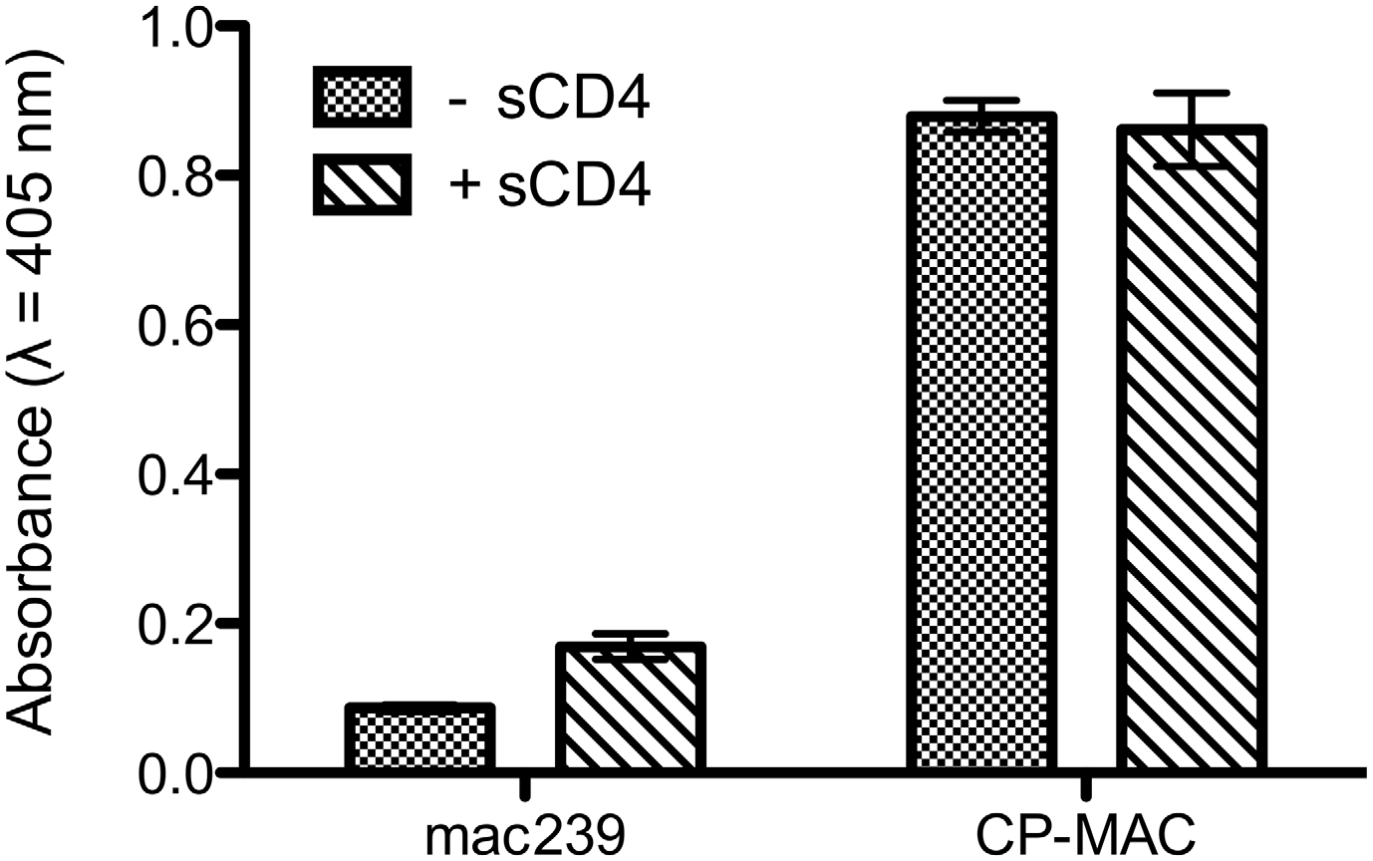
ELISA analysis comparing the binding of 7D3 to either SIVmac239 or SIV CP-MAC viruses. The binding analyses, carried out under the same conditions as the tomographic experiments, show that in contrast to SIVmac239, SIV CP-MAC binds efficiently to 7D3 in the absence and presence of added sCD4-183.

**Figure 7:**
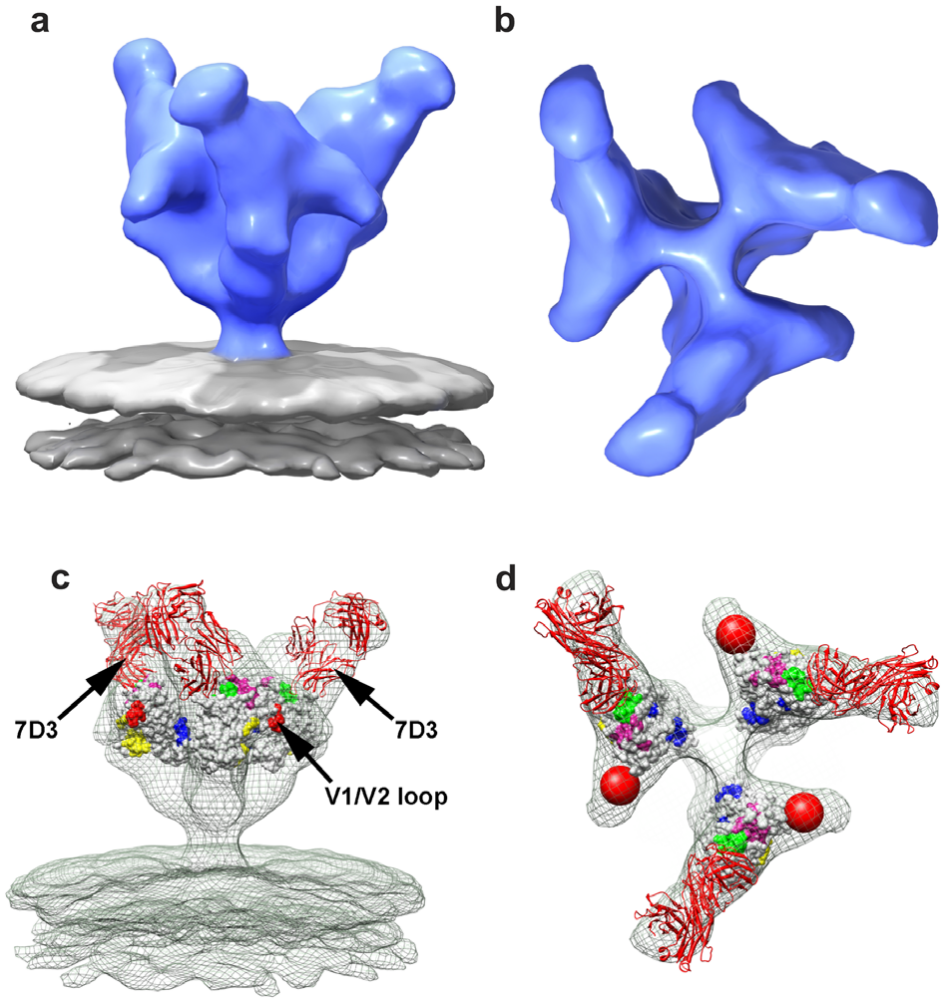
Molecular architecture of co-receptor-binding site antibody (7D3) complexes on SIV CP-MAC Env and fitted gp120 coordinates. (a, b) Perspective (a) and top (b) views of 7D3-bound SIV CP-MAC Env rendered as isosurfaces. (c, d) Fit of gp120 coordinates shown as isosurfaces to the density maps (green mesh) for 7D3-bound SIV CP-MAC Env rendering from perspective (c) and top views (d). The coordinates for 17b Fab were utilized for representing 7D3 (shown in red ribbons) and fit into the difference density calculated between the antibody-bound and corresponding unliganded SIV CP-MAC Env maps. Residues in the CD4 binding site and in the stem of the V1/V2 and V3 loops are shown in yellow, red and green, respectively, with proposed coreceptor-binding site (CoRbs) residues [34] shown in magenta.

The finding that SIV CP-MAC Env displays a quaternary conformation that is similar to that achieved by HIV-1 subsequent to CD4 binding suggests that this should be testable by the structural analysis of complexes formed between SIV CP-MAC and antibodies that recognize potential co-receptor binding site regions. To test the functional relevance of the “open” SIV CP-MAC conformation, we therefore carried out structural analysis of the complex formed with the 7D3 neutralizing antibody, which has been proposed to target co-receptor binding sites on SIV CP-MAC gp120 in a manner similar to that of the CD4i antibody 17b [22], [18]. Whole virion binding studies establish that 7D3 binds much more efficiently to purified SIV CP-MAC than to SIVmac239 under the conditions of the tomography experiments (Figure 6). Cryo-electron tomographic analyses of 7D3 bound viruses show that density for bound antibody is clearly visible on trimeric CP-MAC Env (Figures 7a, 7b) and fitting atomic coordinates for gp120 into 7D3-bound CP-MAC Env density maps shows that 7D3 binds over the base of the V3-loop (Figures 7c, 7d). The overall conformation of the trimeric gp120 itself is unchanged in the complex with 7D3, thus validating the conclusion that SIV CP-MAC Env is in a constitutively open conformation that does not undergo further significant changes upon binding of a co-receptor binding site antibody.

## Discussion

Knowledge of the structure and functionally relevant conformational changes of trimeric Env remains an important challenge in HIV structural biology. While there are X-ray structures reported for selected monomeric gp120 constructs, mostly in ternary complexes with sCD4 and bound Fab fragments, there is a large gap in structural information on the structures of the trimeric gp120 and gp41 as they are displayed in the surface of infectious viruses. Earlier reports of the structure of trimeric SIV Env [13], [14] spawned a controversy [23] that appears attributable to technical problems in data analysis and lack of unambiguous fitting constraints. The results from cryo-electron tomographic analyses of trimeric SIV Env presented here (using wedge-corrected, reference-free image classification methods [10]) address these concerns since the strains used here are the same as those in the previous studies with SIVmac239 [13], and with SIVmneE11S [14]. Comparative analyses of the V1/V2 loop-deleted viral strain further validates the methodology we have employed for structure determination.

**Figure 8:**
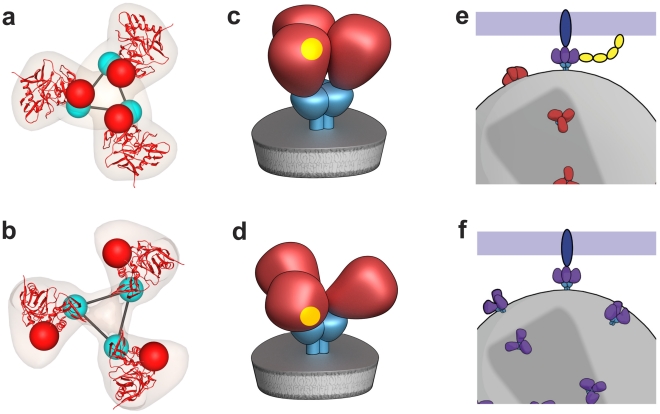
Distinct quaternary conformational states of trimeric Env. (a,b) Top views of fitted density maps for experimentally derived maps of trimeric Env representing the “closed” states of SIVmneE11S (a), and the “open” state of SIV CP-MAC (b). Coordinates for the gp120 core are shown in red ribbons, density maps shown in transparent brown. The likely locations of the gp120/gp41 interface are indicated by linked cyan spheres, whereas V1/V2 loops are indicated by red spheres. (c,d) Schematic view of trimeric envelope glycoprotein spikes showing the closed (c) and open (d) states, with gp120, gp41, and CD4-binding site in red, cyan, and yellow, respectively. (e,f) Schematic comparison of the mechanism of cell-virus contact in CD4-dependent vs. CD4-independent viral entry. For CD4-dependent entry (e), the transition of the spike from the closed (red) to open (purple) state occurs in the presence of CD4 (yellow) and co-receptor (blue) binding, while in CD4-independent entry (f), trimeric Env is already in a conformation capable of binding to co-receptor.

Our analysis of SIV Env using cryo-electron tomography combined with wedge-corrected 3D averaging and classification methods reveals that SIVmneE11S and SIVmac239 have molecular architectures similar to each other, and to that previously determined for HIV-1 BaL [15]. In contrast, SIV CP-MAC, a laboratory-adapted CD4-independent variant of SIV shows an open trimer resembling that previously reported for HIV-1 BaL Env complexed with sCD4 and 17b. An important technical result from our analyses is the demonstration that cryo-electron tomography can be used to distinguish the conformational variability of native Env trimers on intact viruses, establishing the utility of this approach to study Env structural variation in different strains at ∼20 Å resolution (Figure S6). The discovery of the open state in the CD4-independent strain SIV CP-MAC strain immediately suggests how CD4-independent viruses [21] may gain entry into target cells. A schematic summary of the proposed difference between the molecular mechanisms of cellular entry of CD4-dependent and CD4-independent viruses is presented in Figure 8.

The formation of the open state results in exposure of buried regions of gp120 that would normally only be exposed or formed transiently during virus-cell contact. Prior studies with a CD4-independent variant of HIV-1 IIIB demonstrated that in the absence of CD4, gp120 displayed preformed epitopes that overlapped with co-receptor binding sites that are not normally exposed or formed in gp120 from other virus strains until after CD4 binding occurs [24]. Interestingly, CD4-independent strains tend to be neutralization-sensitive [18], [25] and have been isolated from SIV-infected animals with advanced immunodeficiency [26], from immune-privileged areas such as the CNS [27], or upon passage under selection in cell culture [20], [28], [19], [24]. Further, antibodies that react with the exposed epitopes on SIV CP-MAC Env (Figure 5d) that include highly conserved residues such as Asn 262 and Gly 263 have already been identified [18]. 7D3, a potent, neutralizing antibody elicited against CP-MAC Env, binds a conformational epitope thought to reside near the CCR5 binding site [18]. Automated fits of gp120 coordinates to the 7D3-bound CP-MAC Env confirm that the footprint of 7D3 resides over the base of the V3 loop, which is known to interact with co-receptor. These density maps provide a structural foundation for the findings [18], [24] that there is stable exposure of the co-receptor binding site in CD4-independent immunodeficiency viruses. Our results are also consistent with the idea [19], [24], [25] that the joint requirement of CD4 and CCR5 for HIV entry represents an evolutionary adaptation of CCR5-dependent HIV and SIV to use CD4-dependent spike opening as a mechanism to cloak conserved antigenic sites on gp120 that represent targets for neutralizing antibodies until contact occurs on the target cell, where access to antibodies is likely kinetically and sterically restricted. Successful efforts at HIV vaccine design are likely to require a good understanding of Env architecture not only at the level of the structure of a particular trimer or trimer-ligand complex, but of a range of Env-ligand complexes from a variety of SIV, HIV-1 and HIV-2 viral isolates in unliganded, sCD4-liganded and antibody-neutralized states in the physiologically relevant context of an infectious virus. Continuing technological advances in high-resolution cryo-electron microscopy in combination with advanced methods for 3D image processing [9] promise to provide increasingly sophisticated tools to address this challenge.

## Methods

### Sample preparation

SIV and HIV-1 viruses were produced by infection of SupT1 cells, treated with 2,2′dithiodipyridine (Aldrithiol-2, AT-2) and purified by sucrose gradient centrifugation to obtain concentrated preparations (typically ∼10^11^ virions/mL) that retained functionally intact Env. AT-2 treatment renders viruses non-infectious by preferential covalent modification of internal virion proteins required for infectivity, but under conditions known to maintain the conformational and functional integrity of the viral envelope glycoprotein, including full competence for receptor dependent target cell membrane binding and fusion and viral entry [29]. The SIVmneE11S and SIVmac239 virus preparations, provided by the Biological Products Core of the AIDS and Cancer Virus Program, SAIC Frederick, Inc., were identical to those used in the previous conflicting reports of SIV Env structure for SIVmac239 by Zhu et al. [13] and SIVmneE11S by Zanetti et al. [14]. HIV-1 R3A containing a deletion of all but the first and last amino acid of the V1/V2 loop, plus a Gly-Ala-Gly linker, has been previously described [17]. Holey carbon-coated 200 mesh grids for electron microscopy were purchased from Quantifoil GmbH (Jena, Germany) and glow-discharged immediately prior to specimen preparation. 7D3 antibody was purified from mouse ascites fluid with Pierce NAb Spin Kits using Protein G resin, and added to CP-MAC at a concentration of 4 µM and incubated on ice for 30 minutes. Samples were deposited on the grids at room temperature and transferred to the chamber of a Mark III Vitrobot (FEI Company, OR) maintained at 25°C and 100% humidity. Grids were then blotted for 6 sec and plunged into liquid ethane cooled by liquid nitrogen. The total length of time that the specimens were handled at room temperature before plunge-freezing was ∼3–4 minutes.

### Infectivity assay

CD4-independent replication of SIVmac239 and CP-MAC was determined by adding virus (50 ng of p27^gag^) to SupT1 cells or BC7 (a CD4-negative variant of SupT1), that were engineered to stably express human or rhesus CCR5 [30]. Cells were washed after 18 hours to remove exogenous virus and reverse transcriptase levels in culture supernatant determined over time. Infection was also monitored directly by immunofluorescence microscopy on methanol/acetone fixed cells using an anti-p27^gag^ monoclonal antibody (Figure S8).

### Whole virion ELISA

Based on estimated Env concentrations, whole virions were equally coated onto a 96-well plate, sealed and incubated overnight at 4°C. All subsequent steps were performed at 4°C and washes were performed with cold TNE buffer (100 mM Tris, 150 mM NaCl and 1 mM EDTA pH = 7.5). Wells were washed once, blocked with 1% BSA for 1 hour, and washed three more times. Primary 7D3antibody (1 µg/mL) was incubated for 1 hour and washed three times. Goat α-Mouse IgG, H and L chain alkaline phosphatase-conjugated secondary antibody (Calbiochem EMD, 1∶5000 dilution) was incubated for 1 hour and three washes were performed. 4-nitrophenyl phosphate (Sigma-Aldrich, 1 mg/mL) in alkaline phosphatase substrate buffer (50 mM NaHCO_3_, 1 mM MgCl_2_, pH = 9.8) was added at 25°C and absorbance was measured at 405 nm at various time points until saturation. Controls against non-specific binding of primary and secondary antibodies were performed and data were corrected accordingly.

### Data for cryo-electron tomography

Data sets were collected on an energy-filtered (Gatan) Tecnai G2 Polara transmission electron microsocope (FEI, Netherlands) equipped with a 2K×2K post-energy filter CCD camera, operated at 200 kV and with the specimen maintained at −193°C. Data collection was carried out over a tilt range spanning ± 65° with tilt increments ranging from 1–2° and a defocus of 2.5 µm, with pixel sizes of 4.1 Å at the level of the specimen. Doses used for each image were between 1–2 el/Å^2^. Tilt series were aligned using manual fiducial-based alignment as implemented in IMOD [31]. Protein A gold fiducials (10 nm) were selected, tracked and positions refined. Tomograms were then reconstructed using R-weighted back projection. Virion centroids were identified manually and subtomograms (480×480×480 voxels) containing only the virions were selected. The final number of tilt series used for reconstruction for the SIVmneE11S, SIVmac239, SIV CP-MAC and SIV CP-MAC/7D3 maps were 21, 54, 57 and 37, respectively, consisting of 136, 305, 257 and 208 virions respectively, yielding 3800, 4576, 3797 and 1600 putative spikes, respectively.

### Particle picking

For purposes of selection of Env spikes, virion subtomograms were down-sampled by a factor of 4, denoised using edge-enhancing anisotropic diffusion as implemented in IMOD and subjected to unsupervised membrane segmentation using an energy-based three-dimensional approach [32]. In order to identify the location of spikes in an automated manner, a scalar value was associated to every point on the segmented virion surface corresponding to the cross-correlation value between an external 3D template and the image data immediately outside the membrane. Spikes were identified at the locations corresponding to the local maxima of this function that were above a given threshold. A cylindrically symmetric phantom was used as a template for the search; the same template was used for all maps.

### Classification and averaging

Subvolumes (100×100×100 voxels) corresponding to reconstructions of individual spikes (without denoising or binning) were cut from the virion subtomograms at the automatically extracted positions. The orientations of the long axis of the spike were determined using the normal to the automatically segmented membrane at the location of each spike, providing initial estimates for two of the three Euler angles. The remaining in-plane rotation was initially randomized to prevent any possible bias in subsequent alignments. After application of the Euler angles, sub-volumes were translationally aligned to their cylindrically averaged global average to ensure they all shared the same center of mass. The 10% of subvolumes that correlated most poorly with the updated global average were left out of the analysis. Spike volumes were aligned and classified without using external references and with proper accounting of the missing wedge using the framework described in Bartesaghi et al. [10]. Subvolume alignments were progressively refined at each iteration and spike volumes repeatedly clustered into 10 classes. Early stages of classification clearly showed classes with inherent 3-fold symmetry, and typically at the fourth iteration, 3-fold symmetry was imposed. At each round, the classes that showed the most clearly delineated features in all regions of the spike (typically ∼50–60%) were selected and combined to be used as reference for the next round. Typically ∼4000 spikes were selected for each dataset. Final maps were obtained after ∼5–12 refinement rounds and included contributions from ∼50% of sub-volumes in each dataset.

The computational procedures we have used to obtain 3D density maps by averaging tomographic subvolumes were extensively tested first using simulated phantom objects to develop robust alignment and classification routines that take into account the missing wedge data and faithfully recover 3D structures from a series of heterogeneous 3D objects [10]. Experimental assessment of the computational developments were followed by obtaining 3D tomographic models of purified GroEL, a multimeric molecular complex whose structure is known at high resolution from X-ray crystallographic analysis, thereby enabling quantitative evaluation of each map. This computational approach yielded a 26 Å GroEL structure from as few as ∼300 volumes and without imposition of 7-fold symmetry inherent to the GroEL complex [10]. Application of these methods to ∼3000–4000 tomographic volumes of trimeric Env from SIV/HIV-1 strains resulted in maps at a resolution of ∼20 Å (Figure S6). The classes are relatively homogeneous at the end of the refinement as illustrated by inspecting sections through the maps corresponding to each class average (Figure S7). Notably, the computational procedures used by Zhu et al. [13] did not employ procedures to correct for the missing wedge, did not use either reference-free methods for 3D alignment of the individual spike subvolumes, or reference-free methods for image classification. The procedures used by Zanetti et al. [14] used missing wedge correction, but did not use image classification within the iterative alignment procedure.

### Coordinate fitting

Steepest-ascent local optimization, as implemented in UCSF Chimera was utilized for fitting coordinates into density maps [33]. Coordinates were initially placed in random orientations and local maxima of the sum of pointwise products between the coordinates and the map were determined. Fitting was performed to convergence by performing multiples of 100 steepest ascent steps. Atomic coordinates were fit by generating a map simulated from the atomic coordinates at 20 Å. The fits shown in Figure 2–5 and Figure 7 were carried out using 1GC1 coordinates. These coordinates contain a truncated version (residues 119–129, and 194–202) of the V1/V2 loop region (spanning residues 119–202). To eliminate any bias in the fits from the inclusion of the partial loop residues present in the coordinates, they were excluded for purposes of coordinate fitting; even with their inclusion there were no significant changes in the fits. To fit the 7D3 density, the atomic coordinates for 17b were utilized and originally placed in difference density calculated from subtracting the CP-MAC density map from the 7D3-bound CP-MAC map and the 7D3 position was further refined to convergence.

## Supporting Information

Figure S1 Previously reported Env molecular architecture and trimeric models using gp120 coordinates. (a–d) Previously reported density maps for trimeric HIV-1 and SIV Env based on cryo-electron tomography combined with 3D averaging for trimeric Env from (a) SIVmac239 from Zhu et al. (Roux and colleagues 2006; [13]), (b) SIVmneE11S from Zanetti et al. (Fuller and colleagues 2006; [14]), (c) HIV-1 BaL from Zhu et al (Roux and colleagues 2008; [16]) and (d) HIV-1 BaL from our laboratory [15]. All four maps are shown as isosurface representations.
(2.15 MB TIF)

Figure S2 Expanded version of Figure 1d showing slices through the density map of trimeric SIVmneE11S Env at each iteration (from 1 to 7). The slices, spaced by 4.1 Å, are oriented with the bottom slices corresponding to the viral membrane, and top slices corresponding to the apex of the spike. A surface representation of the density map (same as Figure 1f) is presented at right to provide a reference for the orientation of the stack of slices.
(2.36 MB TIF)

Figure S3 Comparison of fits of 1GC1 (a, b) and 2BF1 (c, d) coordinates to the density map for trimeric SIVmneE11S Env. Two thresholds are shown, the lower threshold is more transparent as shown in Figure 1f–1h and the higher threshold is less transparent, highlighting the shape of gp120 density and corresponding coordinate fits. (a, b) Front and top views, respectively, of the fit of the coordinates [7] for gp120 (red ribbons) reported for the complex formed between truncated monomeric HIV-1 gp120, sCD4 and the Fab fragment of 17b to the experimentally derived density map for unliganded SIVmneE11S. These fits were derived by automated fitting of the coordinates to the density map using procedures implemented in the visualization program UCSF Chimera [33]. Other previously reported coordinates for HIV-1 gp120 in the sCD4-liganded state (2B4C and 2NY7) also resulted in similar orientations for gp120 in the density maps with density for the V1/V2 loops at the top of the spike (black arrows). (c, d) Front and top views, respectively, of the fit of the coordinates for gp120 previously reported for unliganded, monomeric SIV gp120 [7] (yellow ribbons) to the experimentally derived density map for unliganded SIVmneE11S. The orientations of gp120 shown to match that presented in the theoretical model proposed by Chen et al. [5] based on their crystallographic structure of unliganded, truncated SIV gp120. In this model, the V1/V2 loop regions were proposed to lie near the outer periphery of the base of the spike.
(2.58 MB TIF)

Figure S4 Fit of gp120 coordinates to density map of the SIVnmeE11S Env spike. Density maps (green transparent isosurface in a, b, c) corresponding to the structures available for the truncated gp120 core (magenta ribbons) were computed at 20 Å resolution and these were fit into the experimentally determined density maps for the native spike using automated fitting functions implemented in the software package Chimera; front (d, e, f) and top (g, h, i) views are shown. The map orientation is identical in panels (a)–(f), and orthogonal to the orientation shown in panels (g)–(i). Visual inspection shows that the shapes of the 1GC1 (a, d, g) and 2NY7 (b, e, h) coordinates follow the shape of the experimentally determined map, while the 2BF1 (c, f, i) coordinates do not show obvious shape complementarity. The red spheres indicate the likely positions of the V1/V2 loop regions based on location of the corresponding truncated loops in the coordinates. In the 1GC1 and 2NY7 coordinates, the estimated location of the V1/V2 loop shows an excellent correspondence to the region of unassigned density at the apex of the spike, while the estimated location of this loop in the 2BF1 coordinates falls in a region of the density map where there is no unassigned density, and is not consistent with the observed architecture of the spike. All three sets of coordinates have significant deletions in the N and C-terminal regions which are expected to reside at the base of the spike, corresponding to the unassigned density visible in the map. As in Figure 2g and 2h, the 2BF1 coordinates were positioned in an orientation that corresponds to the preferred positions suggested by Chen et al. [5].
(3.62 MB TIF)

Figure S5 (a,b) Quantitative estimate of fit of different gp120 coordinates to density maps for SIVmneE11S (a) and SIVmac239 (b) Env by calculation of the number of atoms that are excluded in the map over a range of density thresholds. Using the best fits determined for gp120 from 1GC1 and 2NY7 and Chen's theoretical model for 2BF1 [5], threshold values for density map visualization were progressively varied. At each threshold value, the proportion of atoms that fall outside the map contour was calculated. The plot shows that compared to the fits obtained using 1GC1 or 2NY7 coordinates, a substantially higher proportion of atoms are distributed outside the map contour when 2BF1 coordinates are used to carry out the fits for both SIVmneE11S and SIVmac239 Env.
(0.95 MB TIF)

Figure S6 Fourier Shell Correlation (FSC) plots of the final maps for SIVmneE11S (red), SIVmac239 (green) and SIV CP-MAC (blue). The resolution at which the Fourier shell correlation drops to 0.5 is taken to represent the resolution limit of the density maps. FSC resolution estimates are ∼21 Å for all three maps.
(0.13 MB TIF)

Figure S7 Illustration of class variation at the end of refinement of trimeric Env from SIVmneE11S, SIVmac239 and SIV CP-MAC viruses. Each row represents a class average obtained utilizing ∼ 4000 subvolumes. Sections through the density map of each class average are shown starting from the level of the lipid bilayer membrane (left end) to the top of the spike (right end) for the ten image classes. Each class average is very similar, but the classes with the highest signal-to-noise ratios and closest correlation coefficients (for example, classes 3, 4, 5, 7, 8 and 10 in SIVmneE11S) are averaged together to generate the final 3D averaged maps (bottom row).
(4.64 MB TIF)

Figure S8 Detection of viral antigens on SIV CP-MAC- and SIVmac239- infected cells. BC7/Rh-CCR5 (CD4-negative; rhesus CCR5 positive) and SupT1/Hu-CCR5 (CD4-positive; human CCR5 positive) were inoculated with SIV CP-MAC or SIVmac239 and viral antigens assayed on day 8 by immunofluorescence microscopy with a p27^gag^ monoclonal antibody. Corresponding to the values for reverse transcriptase activity in culture supernatnants (see Figures 4a and 4b), SIV CP-MAC can infect both CD4-positive and -negative cells; SIVmac239 can only infect the CD4-positive SupT1/Hu-CCR5 cells, with only background fluorescence detectable on BC7/Rh-CCR5 cells.
(2.59 MB TIF)

Video S1 Tomographic reconstruction of SIVmneE11S virions displaying envelope glycoproteins.
(8.95 MB WMV)

Video S2 Tomographic reconstruction of SIVmac239 virions displaying envelope glycoproteins (Env).
(10.70 MB WMV)
