# Supplementary figures and images for: Molecular Architectures of Trimeric SIV and HIV-1 Envelope Glycoproteins on Intact Viruses: Strain-Dependent Variation in Quaternary Structure
Source: PLoS Pathog. 2010 Dec 23;6(12):e1001249. doi: 10.1371/journal.ppat.1001249 (PMC3009598; doi:10.1371/journal.ppat.1001249)

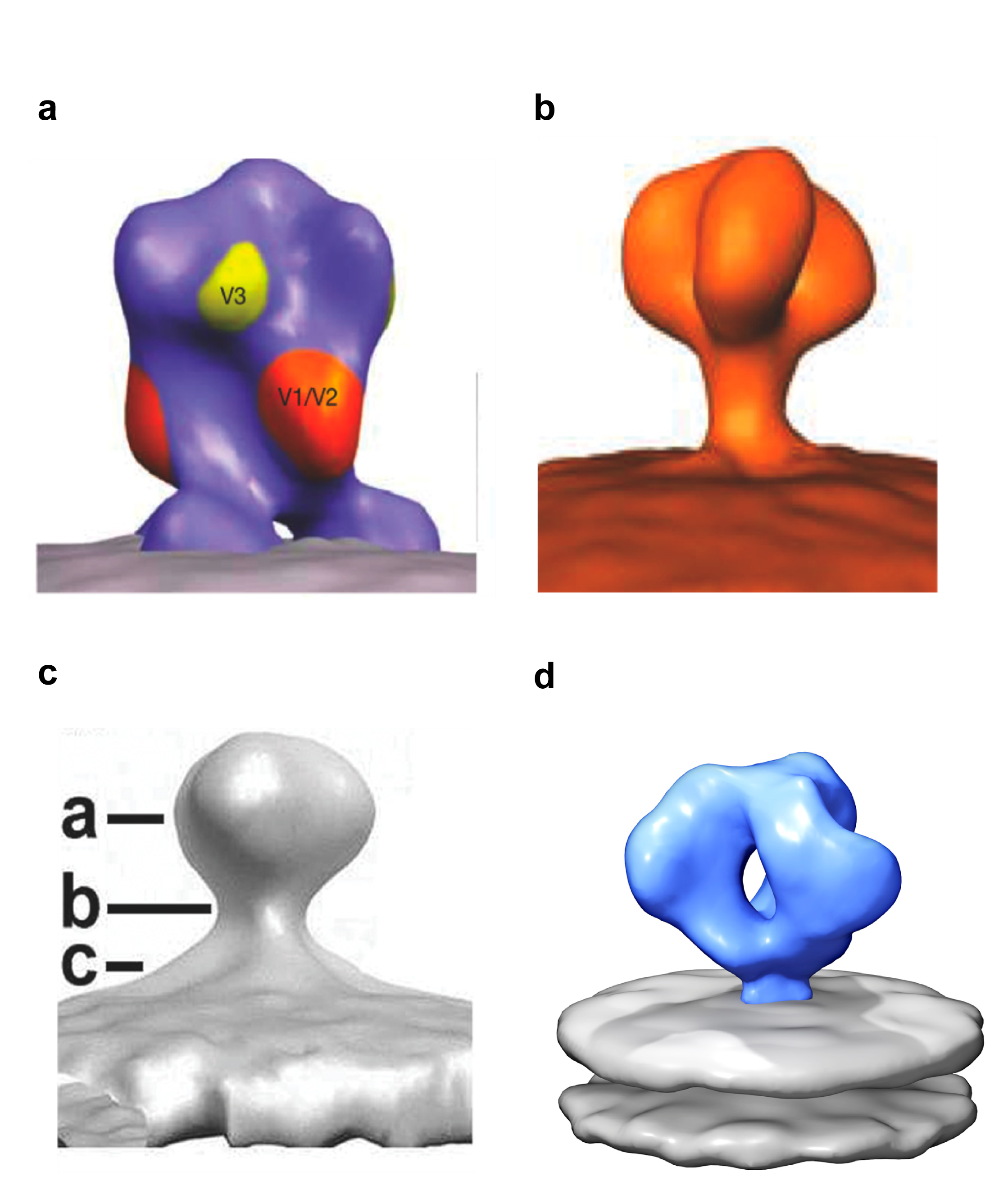

Supplement: Figure S1 — Previously reported Env molecular architecture and trimeric models using gp120 coordinates. (a–d) Previously reported density maps for trimeric HIV-1 and SIV Env based on cryo-electron tomography combined with 3D averaging for trimeric Env from (a) SIVmac239 from Zhu et al. (Roux and colleagues 2006; [13]), (b) SIVmneE11S from Zanetti et al. (Fuller and colleagues 2006; [14]), (c) HIV-1 BaL from Zhu et al (Roux and colleagues 2008; [16]) and (d) HIV-1 BaL from our laboratory [15]. All four maps are shown as isosurface representations. (2.15 MB TIF) [file ppat.1001249.s001.tif]

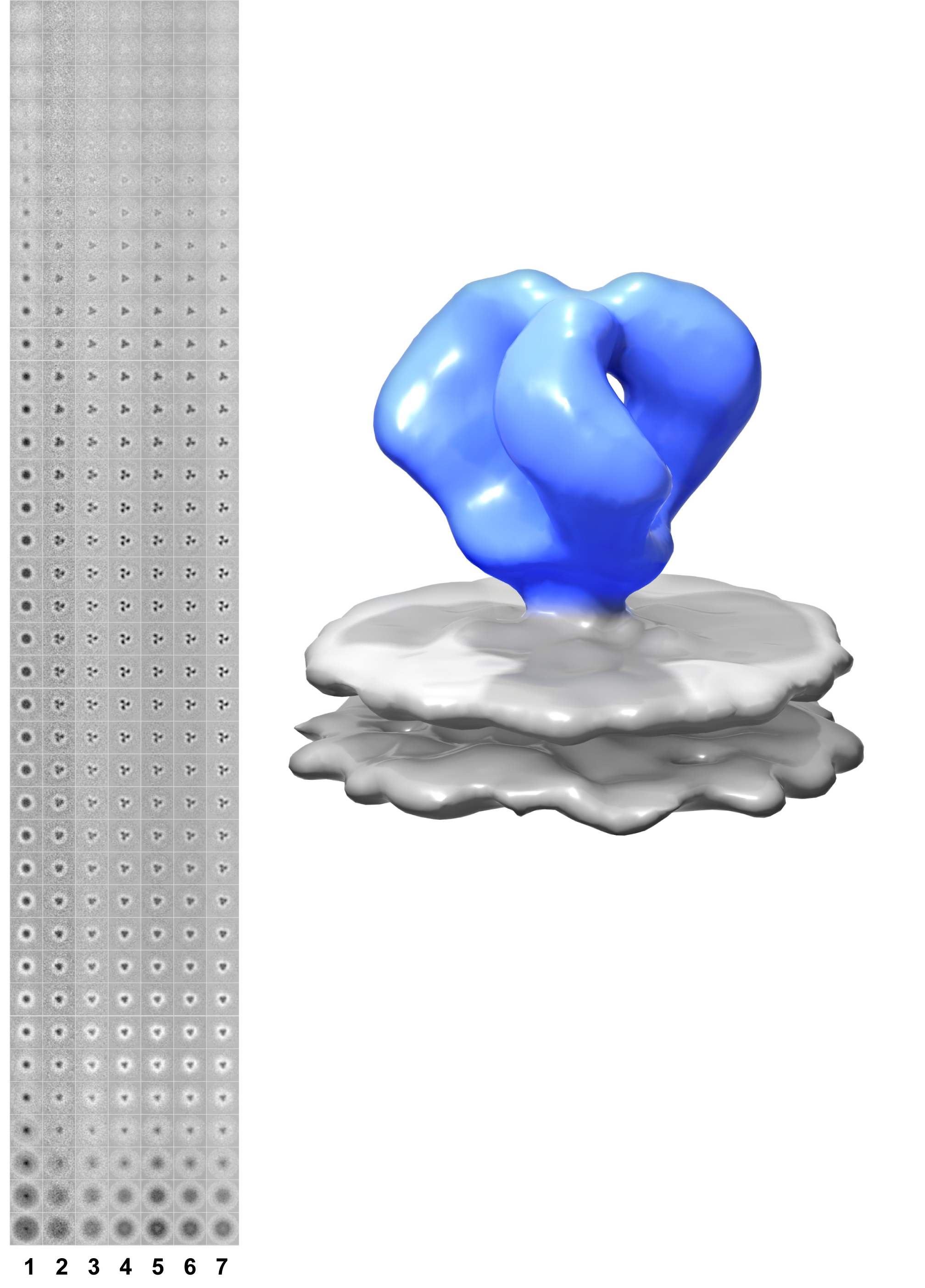

Supplement: Figure S2 — Expanded version of Figure 1d showing slices through the density map of trimeric SIVmneE11S Env at each iteration (from 1 to 7). The slices, spaced by 4.1 Å, are oriented with the bottom slices corresponding to the viral membrane, and top slices corresponding to the apex of the spike. A surface representation of the density map (same as Figure 1f) is presented at right to provide a reference for the orientation of the stack of slices. (2.36 MB TIF) [file ppat.1001249.s002.tif]

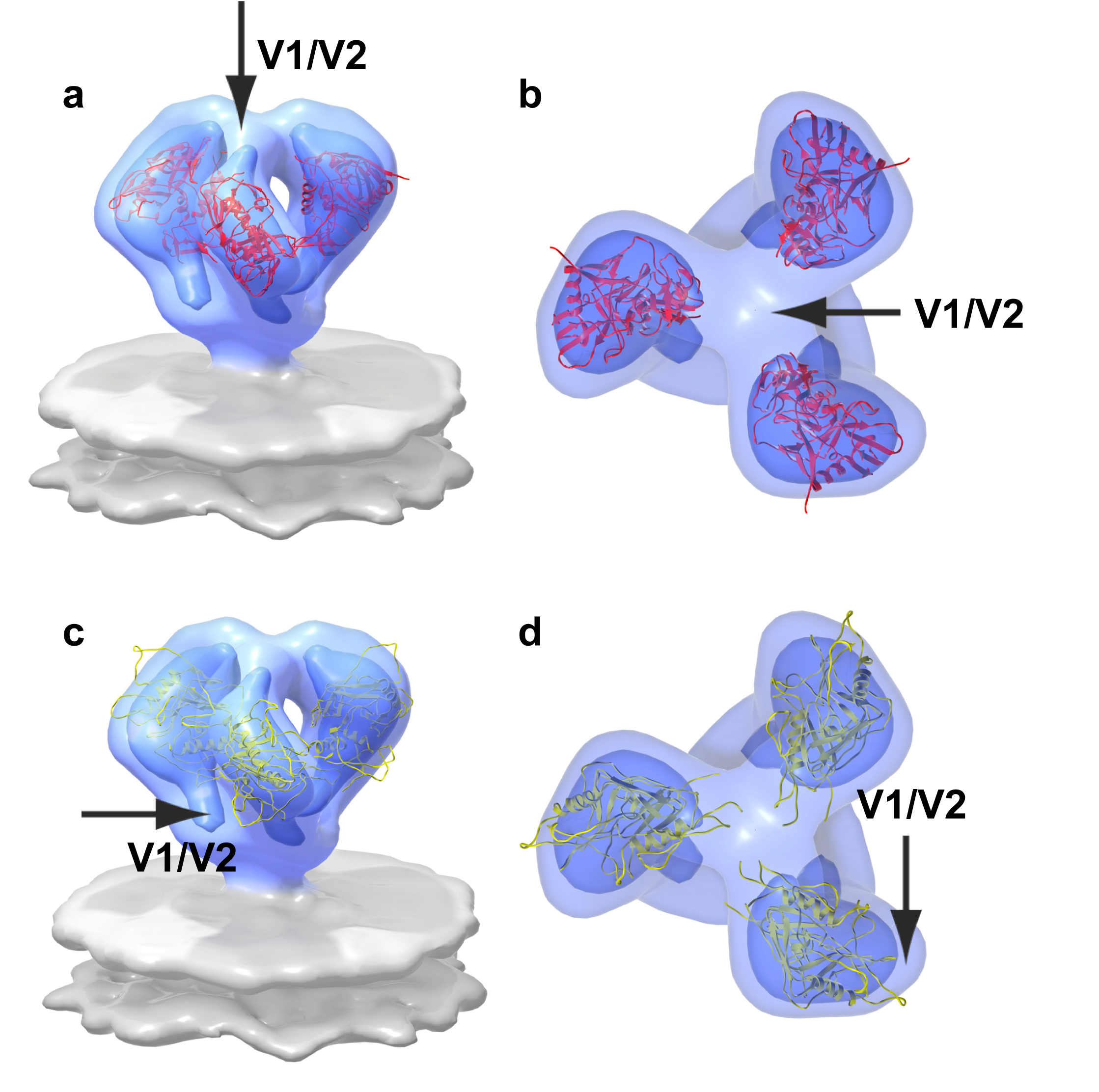

Supplement: Figure S3 — Comparison of fits of 1GC1 (a, b) and 2BF1 (c, d) coordinates to the density map for trimeric SIVmneE11S Env. Two thresholds are shown, the lower threshold is more transparent as shown in Figure 1f–1h and the higher threshold is less transparent, highlighting the shape of gp120 density and corresponding coordinate fits. (a, b) Front and top views, respectively, of the fit of the coordinates [7] for gp120 (red ribbons) reported for the complex formed between truncated monomeric HIV-1 gp120, sCD4 and the Fab fragment of 17b to the experimentally derived density map for unliganded SIVmneE11S. These fits were derived by automated fitting of the coordinates to the density map using procedures implemented in the visualization program UCSF Chimera [33]. Other previously reported coordinates for HIV-1 gp120 in the sCD4-liganded state (2B4C and 2NY7) also resulted in similar orientations for gp120 in the density maps with density for the V1/V2 loops at the top of the spike (black arrows). (c, d) Front and top views, respectively, of the fit of the coordinates for gp120 previously reported for unliganded, monomeric SIV gp120 [7] (yellow ribbons) to the experimentally derived density map for unliganded SIVmneE11S. The orientations of gp120 shown to match that presented in the theoretical model proposed by Chen et al. [5] based on their crystallographic structure of unliganded, truncated SIV gp120. In this model, the V1/V2 loop regions were proposed to lie near the outer periphery of the base of the spike. (2.58 MB TIF) [file ppat.1001249.s003.tif]

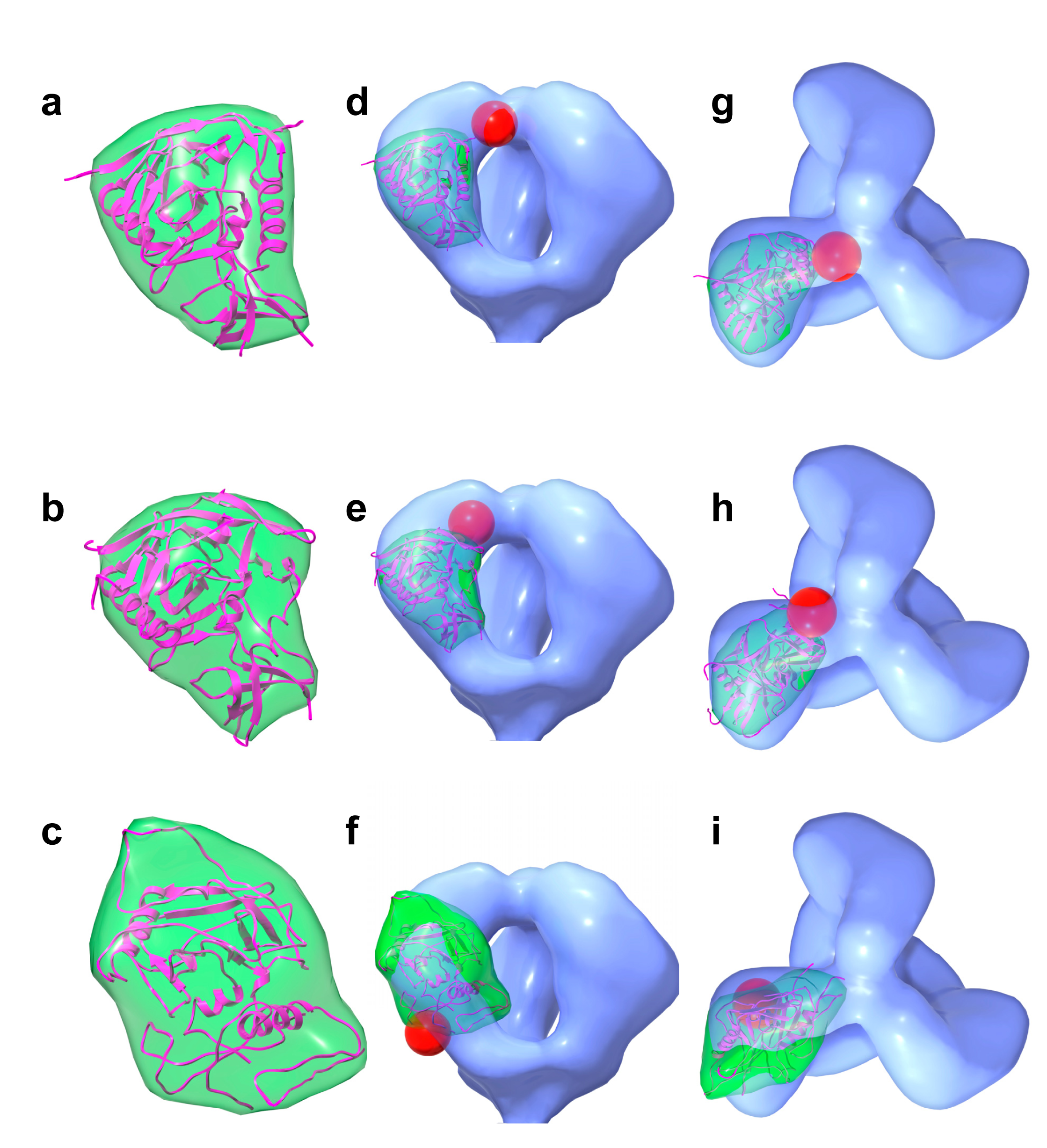

Supplement: Figure S4 — Fit of gp120 coordinates to density map of the SIVnmeE11S Env spike. Density maps (green transparent isosurface in a, b, c) corresponding to the structures available for the truncated gp120 core (magenta ribbons) were computed at 20 Å resolution and these were fit into the experimentally determined density maps for the native spike using automated fitting functions implemented in the software package Chimera; front (d, e, f) and top (g, h, i) views are shown. The map orientation is identical in panels (a)–(f), and orthogonal to the orientation shown in panels (g)–(i). Visual inspection shows that the shapes of the 1GC1 (a, d, g) and 2NY7 (b, e, h) coordinates follow the shape of the experimentally determined map, while the 2BF1 (c, f, i) coordinates do not show obvious shape complementarity. The red spheres indicate the likely positions of the V1/V2 loop regions based on location of the corresponding truncated loops in the coordinates. In the 1GC1 and 2NY7 coordinates, the estimated location of the V1/V2 loop shows an excellent correspondence to the region of unassigned density at the apex of the spike, while the estimated location of this loop in the 2BF1 coordinates falls in a region of the density map where there is no unassigned density, and is not consistent with the observed architecture of the spike. All three sets of coordinates have significant deletions in the N and C-terminal regions which are expected to reside at the base of the spike, corresponding to the unassigned density visible in the map. As in Figure 2g and 2h, the 2BF1 coordinates were positioned in an orientation that corresponds to the preferred positions suggested by Chen et al. [5]. (3.62 MB TIF) [file ppat.1001249.s004.tif]

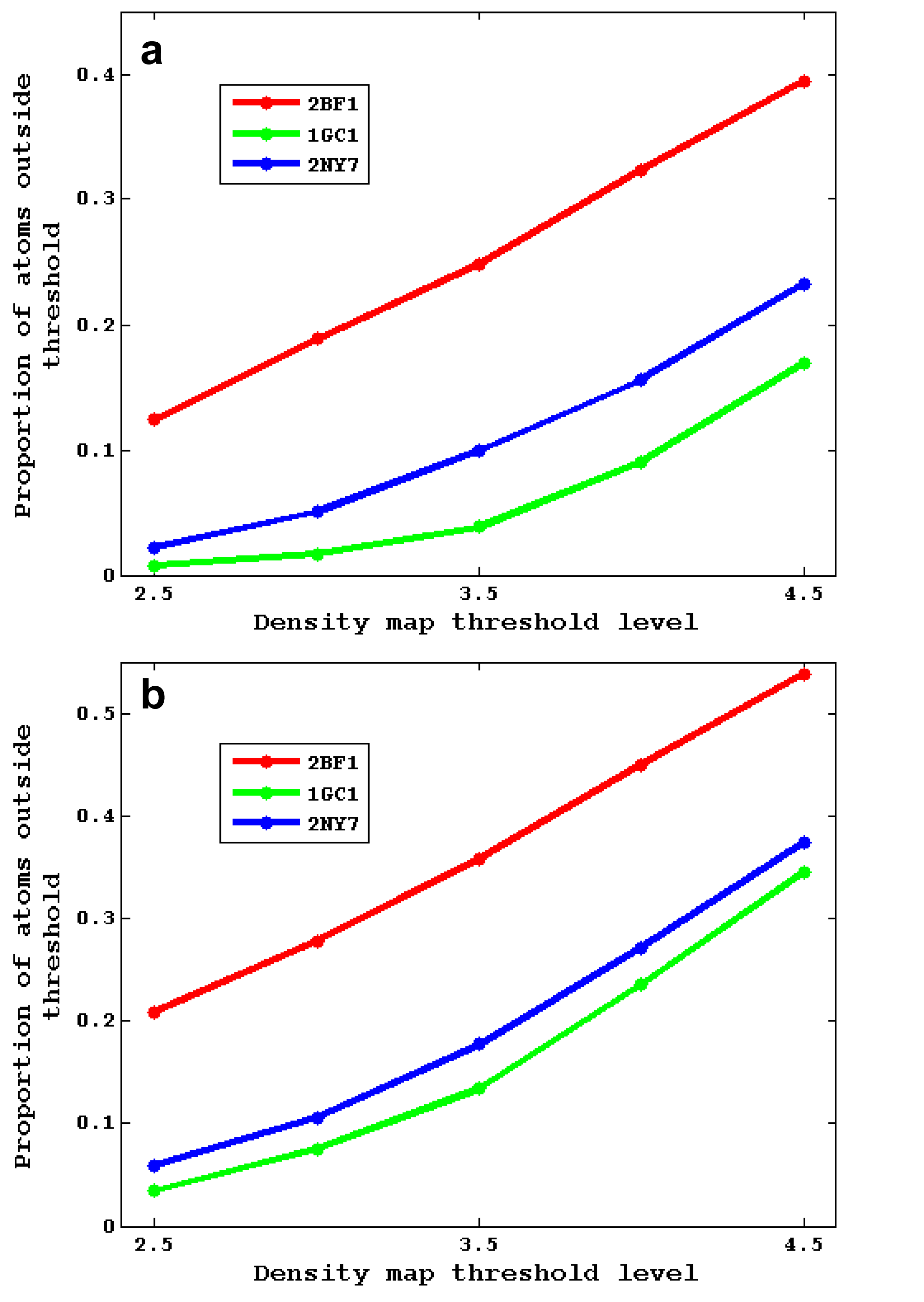

Supplement: Figure S5 — (a,b) Quantitative estimate of fit of different gp120 coordinates to density maps for SIVmneE11S (a) and SIVmac239 (b) Env by calculation of the number of atoms that are excluded in the map over a range of density thresholds. Using the best fits determined for gp120 from 1GC1 and 2NY7 and Chen's theoretical model for 2BF1 [5], threshold values for density map visualization were progressively varied. At each threshold value, the proportion of atoms that fall outside the map contour was calculated. The plot shows that compared to the fits obtained using 1GC1 or 2NY7 coordinates, a substantially higher proportion of atoms are distributed outside the map contour when 2BF1 coordinates are used to carry out the fits for both SIVmneE11S and SIVmac239 Env. (0.95 MB TIF) [file ppat.1001249.s005.tif]

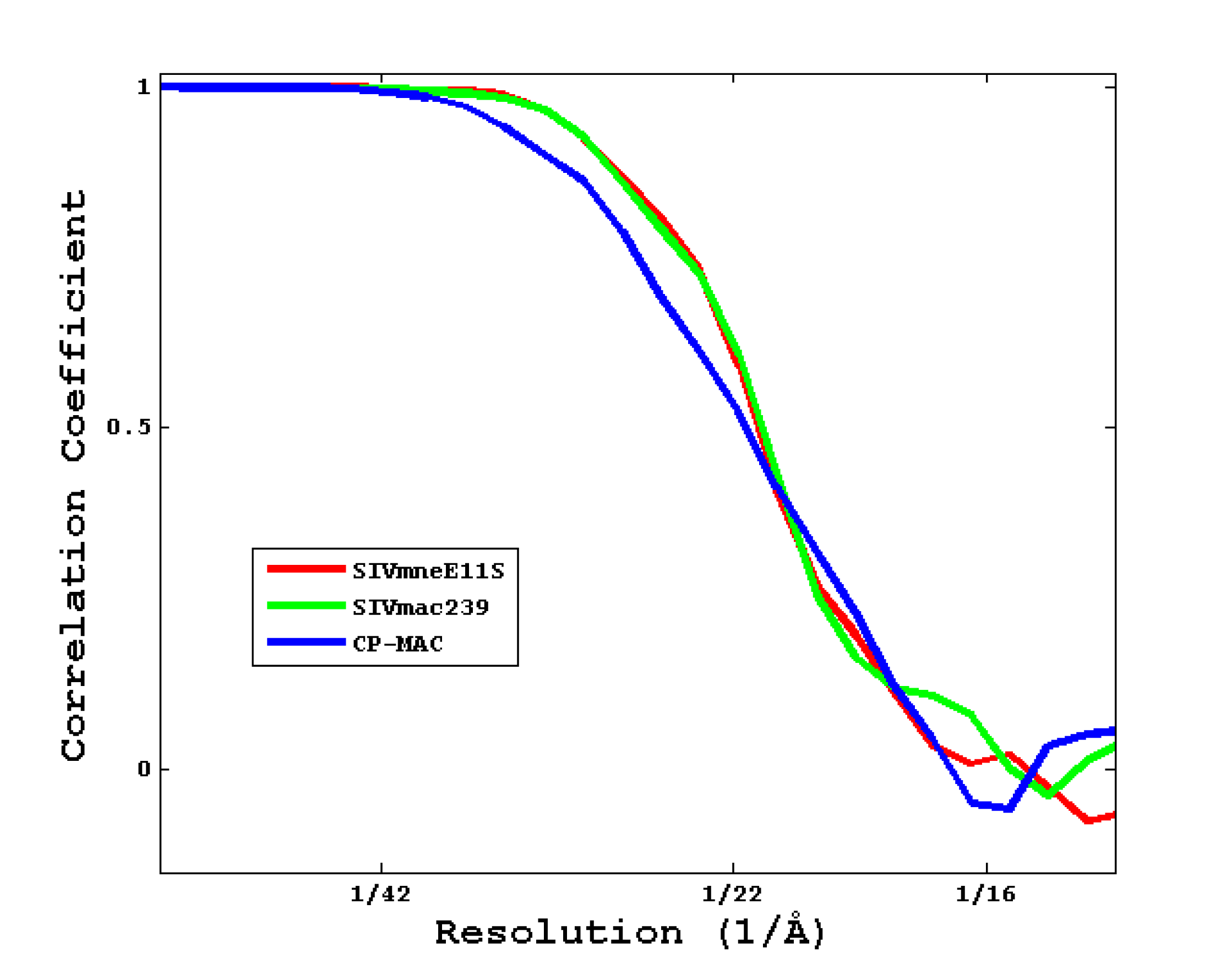

Supplement: Figure S6 — Fourier Shell Correlation (FSC) plots of the final maps for SIVmneE11S (red), SIVmac239 (green) and SIV CP-MAC (blue). The resolution at which the Fourier shell correlation drops to 0.5 is taken to represent the resolution limit of the density maps. FSC resolution estimates are ∼21 Å for all three maps. (0.13 MB TIF) [file ppat.1001249.s006.tif]

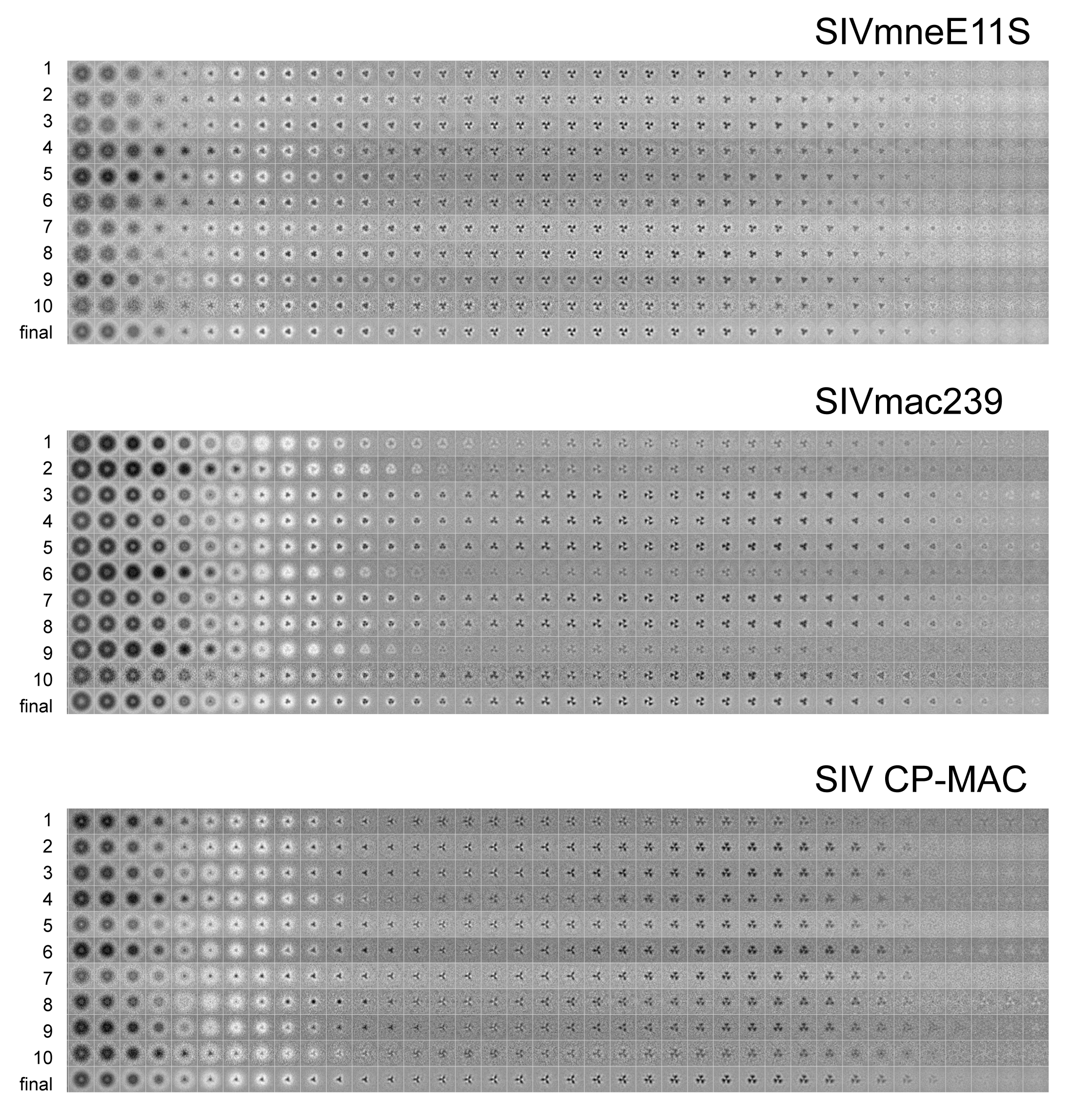

Supplement: Figure S7 — Illustration of class variation at the end of refinement of trimeric Env from SIVmneE11S, SIVmac239 and SIV CP-MAC viruses. Each row represents a class average obtained utilizing ∼ 4000 subvolumes. Sections through the density map of each class average are shown starting from the level of the lipid bilayer membrane (left end) to the top of the spike (right end) for the ten image classes. Each class average is very similar, but the classes with the highest signal-to-noise ratios and closest correlation coefficients (for example, classes 3, 4, 5, 7, 8 and 10 in SIVmneE11S) are averaged together to generate the final 3D averaged maps (bottom row). (4.64 MB TIF) [file ppat.1001249.s007.tif]

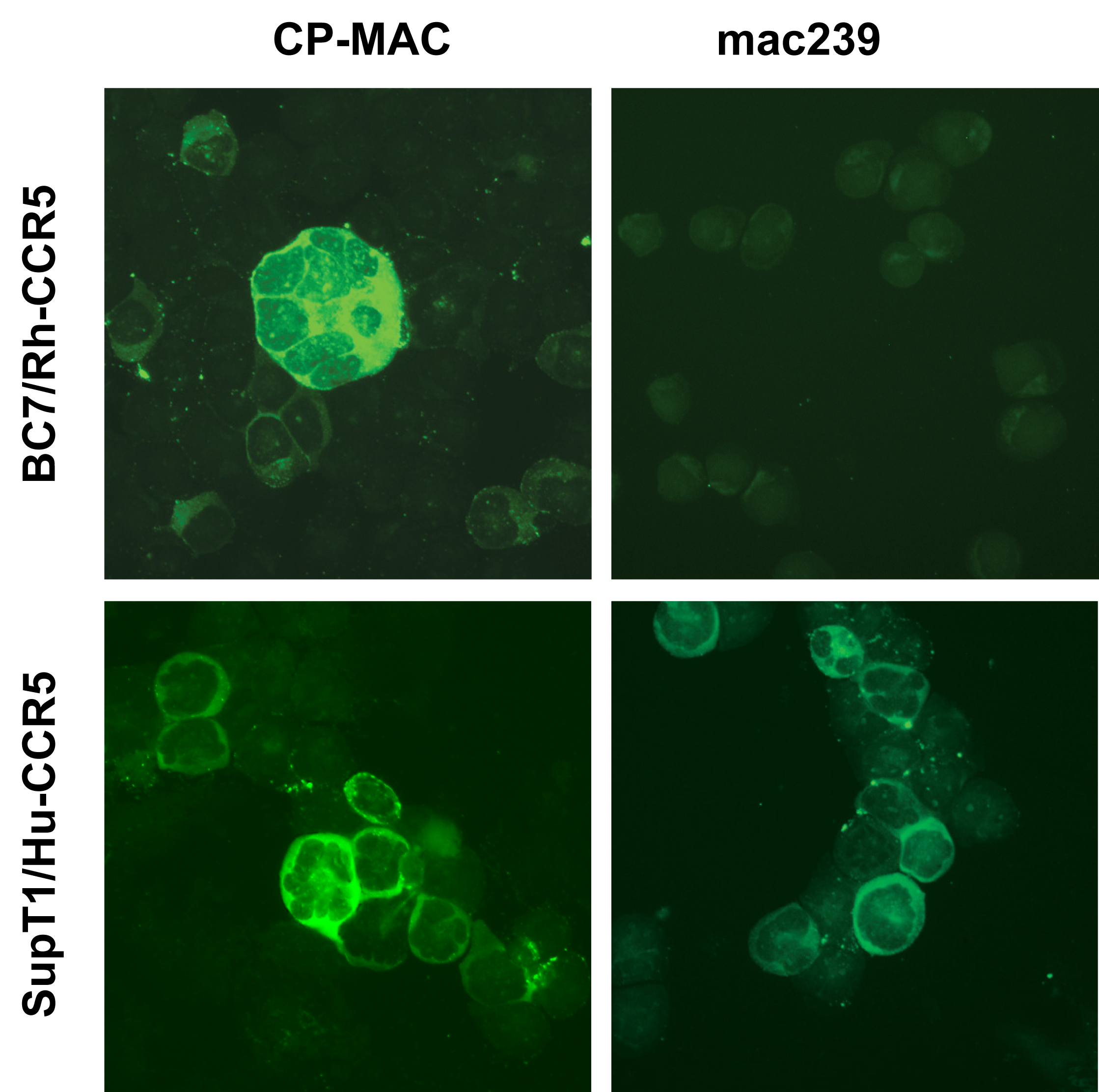

Supplement: Figure S8 — Detection of viral antigens on SIV CP-MAC- and SIVmac239- infected cells. BC7/Rh-CCR5 (CD4-negative; rhesus CCR5 positive) and SupT1/Hu-CCR5 (CD4-positive; human CCR5 positive) were inoculated with SIV CP-MAC or SIVmac239 and viral antigens assayed on day 8 by immunofluorescence microscopy with a p27gag monoclonal antibody. Corresponding to the values for reverse transcriptase activity in culture supernatnants (see Figures 4a and 4b), SIV CP-MAC can infect both CD4-positive and -negative cells; SIVmac239 can only infect the CD4-positive SupT1/Hu-CCR5 cells, with only background fluorescence detectable on BC7/Rh-CCR5 cells. (2.59 MB TIF) [file ppat.1001249.s008.tif]
